# Supplementary material for: Real-world safety and effectiveness of rivaroxaban using Japan-specific dosage during long-term follow-up in patients with atrial fibrillation: XAPASS
Source: PLoS One. 2021 Jun 11;16(6):e0251325. doi: 10.1371/journal.pone.0251325 (PMC8195353; doi:10.1371/journal.pone.0251325)
Supplement: S1 Table — (DOCX) [file pone.0251325.s002.docx]

**S1 Table.** **Incidences of safety outcomes in patient subgroups.**

| **Patient population** | **N** | **Major bleeding** | | **Intracranial hemorrhage** | | **Any bleeding** | |
| --- | --- | --- | --- | --- | --- | --- | --- |
|  |  | **Crude incidence, n** | **Incidence, events per 100 patient-years** | **Crude incidence, n** | **Incidence, events per 100 patient-years** | **Crude incidence, n** | **Incidence, events per 100 patient-years** |
| Overall | 10,664 | 307 | 1.16 | 123 | 0.46 | 961 | 3.77 |
| Age, years |  |  |  |  |  |  |  |
| <65 | 1911 | 29 | 0.59 | 13 | 0.26 | 109 | 2.28 |
| <75 | 5466 | 124 | 0.86 | 52 | 0.36 | 439 | 3.19 |
| ≥65 | 8753 | 278 | 1.28 | 110 | 0.50 | 854 | 4.11 |
| ≥75 | 5198 | 183 | 1.50 | 71 | 0.58 | 524 | 4.45 |
| ≥85 | 1141 | 48 | 2.31 | 16 | 0.77 | 120 | 5.96 |
| Sex |  |  |  |  |  |  |  |
| Male | 6600 | 187 | 1.14 | 80 | 0.48 | 598 | 3.78 |
| Female | 4064 | 120 | 1.19 | 43 | 0.42 | 365 | 3.75 |
| Body weight, kg | | | | | | | |
| ≤50 | 2057 | 69 | 1.55 | 31 | 0.69 | 199 | 4.62 |
| >50 | 7865 | 218 | 1.07 | 85 | 0.41 | 703 | 3.59 |
| BMI, kg/m^2^ |  |  |  |  |  |  |  |
| <18.5 | 589 | 18 | 1.56 | 7 | 0.60 | 63 | 5.75 |
| 18.5 to <25 | 4931 | 146 | 1.18 | 64 | 0.51 | 477 | 4.03 |
| 25 to <30 | 2403 | 71 | 1.11 | 28 | 0.44 | 207 | 3.38 |
| ≥30 | 551 | 11 | 0.73 | 2 | 0.13 | 36 | 2.46 |
| Creatinine clearance, mL/min | | | | | | | |
| <15 | 3 | 0 | 0 | 0 | 0 | 0 | 0 |
| 15 to <30 | 294 | 14 | 3.05 | 5 | 1.07 | 38 | 8.60 |
| 30 to <50 | 2233 | 89 | 1.79 | 35 | 0.70 | 243 | 5.05 |
| 50 to <80 | 4569 | 118 | 0.99 | 50 | 0.42 | 415 | 3.63 |
| ≥80 | 2727 | 65 | 0.89 | 25 | 0.34 | 200 | 2.85 |
| CHADS_2_ score | | | | | | | |
| 0 | 900 | 16 | 0.73 | 8 | 0.36 | 62 | 2.94 |
| 1 | 2601 | 47 | 0.68 | 11 | 0.16 | 208 | 3.12 |
| 2 | 3218 | 85 | 1.01 | 37 | 0.44 | 297 | 3.67 |
| 3 | 2093 | 84 | 1.69 | 34 | 0.68 | 200 | 4.16 |
| 4 | 1261 | 48 | 1.71 | 26 | 0.92 | 124 | 4.59 |
| 5 | 487 | 19 | 1.89 | 5 | 0.49 | 56 | 5.80 |
| 6 | 104 | 8 | 3.44 | 2 | 0.83 | 16 | 7.21 |
| CHA_2_DS_2_-VASc score | | | | | | | |
| 0 | 275 | 2 | 0.32 | 2 | 0.32 | 8 | 1.32 |
| 1 | 1002 | 20 | 0.77 | 6 | 0.23 | 80 | 3.19 |
| 2 | 1807 | 29 | 0.61 | 10 | 0.21 | 135 | 2.96 |
| 3 | 2498 | 65 | 0.99 | 27 | 0.41 | 228 | 3.61 |
| 4 | 2375 | 78 | 1.30 | 33 | 0.55 | 214 | 3.71 |
| 5 | 1566 | 61 | 1.68 | 26 | 0.71 | 169 | 4.85 |
| 6 | 786 | 32 | 1.86 | 13 | 0.75 | 82 | 4.96 |
| 7 | 298 | 15 | 2.60 | 5 | 0.86 | 36 | 6.44 |
| 8 | 54 | 5 | 4.66 | 1 | 0.89 | 11 | 11.15 |
| 9 | 3 | 0 | 0 | 0 | 0 | 0 | 0 |
| Modified HAS-BLED score^a^ | | | | | | | |
| 0 | 1359 | 23 | 0.65 | 5 | 0.14 | 81 | 2.35 |
| 1 | 4523 | 99 | 0.83 | 43 | 0.36 | 349 | 3.04 |
| 2 | 3270 | 111 | 1.41 | 44 | 0.55 | 350 | 4.65 |
| 3 | 1231 | 60 | 2.26 | 26 | 0.97 | 143 | 5.64 |
| 4 | 250 | 9 | 1.61 | 3 | 0.53 | 30 | 5.70 |
| 5 | 29 | 5 | 11.43 | 2 | 4.33 | 10 | 26.18 |
| 6 | 1 | 0 | 0 | 0 | 0 | 0 | 0 |
| 7 | 0 | – | – | – | – | – | – |
| 8 | 0 | – | – | – | – | – | – |
| Baseline comorbidities | | | | | | | |
| Congestive heart failure | 2685 | 97 | 1.54 | 27 | 0.43 | 280 | 4.66 |
| Hypertension | 8007 | 247 | 1.21 | 100 | 0.49 | 753 | 3.85 |
| Diabetes mellitus | 2431 | 93 | 1.52 | 38 | 0.61 | 225 | 3.80 |
| Prior ischemic stroke/TIA | 2450 | 87 | 1.59 | 43 | 0.78 | 238 | 4.52 |
| Vascular disease^b^ | 429 | 24 | 2.44 | 9 | 0.91 | 70 | 7.56 |
| Hepatic dysfunction | 722 | 27 | 1.46 | 8 | 0.43 | 89 | 5.12 |
| Type of AF |  |  |  |  |  |  |  |
| Paroxysmal | 3590 | 93 | 1.07 | 39 | 0.45 | 333 | 4.03 |
| Persistent | 3822 | 107 | 1.13 | 46 | 0.48 | 315 | 3.42 |
| Permanent | 2605 | 90 | 1.30 | 28 | 0.40 | 265 | 3.99 |
| Oral antiplatelet use | 474 | 23 | 2.19 | 13 | 1.24 | 79 | 8.11 |

^a^ Maximum score is 8 because the labile international normalized ratio was excluded.

^b^ Vascular disease is defined as myocardial infarction and/or peripheral artery disease and/or aortic plaque.

Abbreviations: AF, atrial fibrillation; BMI, body mass index; CHADS_2_, Congestive heart failure, Hypertension, Age ≥75 years, Diabetes mellitus, previous Stroke/TIA (2 points); CHA_2_DS_2_-VASc, Congestive heart failure, Hypertension, Age (65–74 years, 1 point; ≥75 years, 2 points), Diabetes mellitus, previous Stroke/TIA (2 points), Vascular disease and female sex; Modified HAS-BLED, Hypertension, Abnormal renal or liver function, previous Stroke, previous major or predisposition to Bleeding, Labile international normalized ratio (excluded from this analysis), Elderly (>65 years), medication use predisposing to bleeding, and previous Drug or alcohol use; TIA, transient ischemic attack.
