# Supplementary material for: Real-world safety and effectiveness of rivaroxaban using Japan-specific dosage during long-term follow-up in patients with atrial fibrillation: XAPASS
Source: PLoS One. 2021 Jun 11;16(6):e0251325. doi: 10.1371/journal.pone.0251325 (PMC8195353; doi:10.1371/journal.pone.0251325)
Supplement: S2 Table — (DOCX) [file pone.0251325.s003.docx]

**S2 Table**. **Adverse events leading to death in the XAPASS.**

| **Events leading to death** | **Patients, n (%)**  **N = 475** |
| --- | --- |
| Bleeding events |  |
| Bleeding | 33 (6.9) |
| Intracranial hemorrhage | 19 (4.0) |
| Extracranial hemorrhage | 14 (2.9) |
| Events classified by organ^a^ |  |
| Cancer^b^ | 97 (20.4) |
| Infections^c^ | 84 (17.9) |
| Cardiac disorders | 82 (17.3) |
| Respiratory disorders^d^ | 63 (13.3) |
| Nervous system disorders | 42 (8.8) |
| Metabolism and nutrition disorders | 27 (5.7) |
| Gastrointestinal disorders | 18 (3.8) |
| Injury, poisoning, and procedural complications | 10 (2.1) |
| Hepatobiliary disorders | 10 (2.1) |
| Others | 79 (16.6) |

Multiple adverse events were reported in some patients.

^a^ Classification is based on system organ class in the Medical Dictionary Regulatory Activities (MedDRA), version 20.0.

^b^ The terms used in MedDRA are cancer, neoplasms, benign, malignant, and unspecified (including cysts and polyps).

^c^ The terms used in MedDRA are infections and infestations.

^d^ The terms used in MedDRA are respiratory, thoracic, and mediastinal disorders.
