# Supplementary material for: Real-world safety and effectiveness of rivaroxaban using Japan-specific dosage during long-term follow-up in patients with atrial fibrillation: XAPASS
Source: PLoS One. 2021 Jun 11;16(6):e0251325. doi: 10.1371/journal.pone.0251325 (PMC8195353; doi:10.1371/journal.pone.0251325)
Supplement: S3 Table — (DOCX) [file pone.0251325.s004.docx]

**S3 Table.** **All adverse events during the standard observation period in the XAPASS.**

|  | **Adverse events** | **Patients, n (%) N = 10,664** | |
| --- | --- | --- | --- |
| Infections and infestations | | 302 | (2.8) |
|  | Appendicitis | 2 | (<0.1) |
|  | Bacteremia | 1 | (<0.1) |
|  | Body tinea | 2 | (<0.1) |
|  | Bronchitis | 28 | (0.3) |
|  | Cellulitis | 10 | (<0.1) |
|  | Conjunctivitis | 1 | (<0.1) |
|  | Cystitis | 11 | (0.1) |
|  | Diabetic gangrene | 1 | (<0.1) |
|  | Disseminated tuberculosis | 1 | (<0.1) |
|  | Diverticulitis intestinal hemorrhagic | 1 | (<0.1) |
|  | Erysipelas | 1 | (<0.1) |
|  | Gangrene | 1 | (<0.1) |
|  | Gastroenteritis | 7 | (<0.1) |
|  | Gastroenteritis staphylococcal | 1 | (<0.1) |
|  | Herpes zoster | 12 | (0.1) |
|  | Infection | 1 | (<0.1) |
|  | Influenza | 9 | (<0.1) |
|  | Liver abscess | 2 | (<0.1) |
|  | Meningitis viral | 1 | (<0.1) |
|  | Nasopharyngitis | 13 | (0.1) |
|  | Otitis externa | 1 | (<0.1) |
|  | Periodontitis | 3 | (<0.1) |
|  | Peritonitis | 2 | (<0.1) |
|  | Pharyngitis | 7 | (<0.1) |
|  | Pneumonia | 140 | (1.3) |
|  | Pneumonia influenzal | 1 | (<0.1) |
|  | Pseudomembranous colitis | 1 | (<0.1) |
|  | Pulmonary tuberculosis | 1 | (<0.1) |
|  | Pyelonephritis | 6 | (<0.1) |
|  | Pyelonephritis acute | 3 | (<0.1) |
|  | Rhinitis | 3 | (<0.1) |
|  | Sepsis | 7 | (<0.1) |
|  | Sinusitis | 1 | (<0.1) |
|  | Skin infection | 1 | (<0.1) |
|  | Subcutaneous abscess | 1 | (<0.1) |
|  | Tinea pedis | 1 | (<0.1) |
|  | Tonsillitis | 3 | (<0.1) |
|  | Tuberculous pleurisy | 1 | (<0.1) |
|  | Upper respiratory tract infection | 4 | (<0.1) |
|  | Urinary tract infection | 19 | (0.2) |
|  | Arthritis bacterial | 2 | (<0.1) |
|  | Helicobacter infection | 2 | (<0.1) |
|  | Wound sepsis | 1 | (<0.1) |
|  | Enteritis infectious | 2 | (<0.1) |
|  | Tinea infection | 2 | (<0.1) |
|  | Pneumonia bacterial | 12 | (0.1) |
|  | Atypical mycobacterial infection | 1 | (<0.1) |
|  | Soft tissue infection | 1 | (<0.1) |
|  | Infective spondylitis | 1 | (<0.1) |
|  | Herpes zoster oticus | 1 | (<0.1) |
|  | Device related infection | 2 | (<0.1) |
|  | Enterocolitis bacterial | 1 | (<0.1) |
|  | Infectious pleural effusion | 1 | (<0.1) |
|  | *Pneumocystis jirovecii* pneumonia | 1 | (<0.1) |
|  | Cholangitis infective | 1 | (<0.1) |
| Neoplasms benign, malignant, and unspecified (including cysts and polyps) | | 262 | (2.5) |
|  | Acute myeloid leukemia | 1 | (<0.1) |
|  | Bile duct cancer | 5 | (<0.1) |
|  | Bladder cancer | 10 | (<0.1) |
|  | Bladder cancer recurrent | 1 | (<0.1) |
|  | Bladder cancer stage 0, with cancer *in situ* | 1 | (<0.1) |
|  | Bladder neoplasm | 3 | (<0.1) |
|  | Bone cancer | 1 | (<0.1) |
|  | Bone neoplasm | 1 | (<0.1) |
|  | Breast cancer | 5 | (<0.1) |
|  | Cholangiocarcinoma | 2 | (<0.1) |
|  | Colon cancer | 14 | (0.1) |
|  | Gallbladder cancer | 2 | (<0.1) |
|  | Gastric cancer | 39 | (0.4) |
|  | Gastric cancer recurrent | 2 | (<0.1) |
|  | Hypopharyngeal cancer | 1 | (<0.1) |
|  | Laryngeal cancer | 2 | (<0.1) |
|  | Leukemia | 1 | (<0.1) |
|  | Liposarcoma | 1 | (<0.1) |
|  | Lung adenocarcinoma | 4 | (<0.1) |
|  | Lung adenocarcinoma recurrent | 1 | (<0.1) |
|  | Lung carcinoma cell type unspecified recurrent | 1 | (<0.1) |
|  | Lung squamous cell carcinoma stage II | 1 | (<0.1) |
|  | Lymphoma | 12 | (0.1) |
|  | Malignant ascites | 1 | (<0.1) |
|  | Malignant pleural effusion | 2 | (<0.1) |
|  | Metastases to bone | 2 | (<0.1) |
|  | Metastases to liver | 4 | (<0.1) |
|  | Metastases to lung | 3 | (<0.1) |
|  | Metastases to lymph nodes | 1 | (<0.1) |
|  | Metastases to pleura | 1 | (<0.1) |
|  | Myelodysplastic syndrome | 3 | (<0.1) |
|  | Nasal sinus cancer | 1 | (<0.1) |
|  | Neoplasm malignant | 1 | (<0.1) |
|  | Neoplasm skin | 2 | (<0.1) |
|  | Non-Hodgkin's lymphoma | 1 | (<0.1) |
|  | Esophageal carcinoma | 10 | (<0.1) |
|  | Ovarian cancer | 2 | (<0.1) |
|  | Pancreatic carcinoma | 18 | (0.2) |
|  | Papillary thyroid cancer | 1 | (<0.1) |
|  | Pharyngeal cancer | 2 | (<0.1) |
|  | Plasma cell myeloma | 2 | (<0.1) |
|  | Polycythemia vera | 1 | (<0.1) |
|  | Rectal cancer | 8 | (<0.1) |
|  | Rectal cancer recurrent | 1 | (<0.1) |
|  | Rectal cancer stage III | 1 | (<0.1) |
|  | Renal cancer | 2 | (<0.1) |
|  | Renal cancer recurrent | 1 | (<0.1) |
|  | Seborrheic keratosis | 1 | (<0.1) |
|  | Skin cancer | 2 | (<0.1) |
|  | Small cell lung cancer | 4 | (<0.1) |
|  | Ureteric cancer metastatic | 1 | (<0.1) |
|  | Uterine cancer | 4 | (<0.1) |
|  | Rectal adenoma | 1 | (<0.1) |
|  | Colon adenoma | 1 | (<0.1) |
|  | Tumor hemorrhage | 11 | (0.1) |
|  | Cardiac neoplasm unspecified | 1 | (<0.1) |
|  | Oral fibroma | 1 | (<0.1) |
|  | Metastases to peritoneum | 1 | (<0.1) |
|  | Small intestine carcinoma | 2 | (<0.1) |
|  | Rectal cancer metastatic | 1 | (<0.1) |
|  | Thyroid cancer metastatic | 1 | (<0.1) |
|  | Hepatic cancer metastatic | 1 | (<0.1) |
|  | Biliary cancer metastatic | 1 | (<0.1) |
|  | Neoplasm of appendix | 1 | (<0.1) |
|  | Lung neoplasm malignant | 34 | (0.3) |
|  | Natural killer-cell lymphoblastic lymphoma | 1 | (<0.1) |
|  | Metastases to central nervous system | 2 | (<0.1) |
|  | Prostate cancer | 13 | (0.1) |
|  | Brain neoplasm | 3 | (<0.1) |
|  | Salivary gland neoplasm | 1 | (<0.1) |
|  | Salivary gland cancer | 1 | (<0.1) |
|  | Gastric cancer stage IV | 1 | (<0.1) |
|  | Gastric neoplasm | 1 | (<0.1) |
|  | Lung neoplasm | 2 | (<0.1) |
|  | Pharyngeal neoplasm | 1 | (<0.1) |
|  | Metastatic gastric cancer | 1 | (<0.1) |
|  | Thyroid cancer | 2 | (<0.1) |
|  | Melanoma recurrent | 1 | (<0.1) |
|  | Renal cell carcinoma | 2 | (<0.1) |
|  | Pericardial mesothelioma malignant | 1 | (<0.1) |
|  | Hepatic cancer | 4 | (<0.1) |
|  | Hepatocellular carcinoma | 2 | (<0.1) |
|  | Gallbladder neoplasm | 1 | (<0.1) |
| Blood and lymphatic system disorders | | 164 | (1.5) |
|  | Agranulocytosis | 1 | (<0.1) |
|  | Anemia | 113 | (1.1) |
|  | Anemia macrocytic | 1 | (<0.1) |
|  | Aplastic anemia | 1 | (<0.1) |
|  | Disseminated intravascular coagulation | 6 | (<0.1) |
|  | Eosinophilia | 1 | (<0.1) |
|  | Febrile neutropenia | 1 | (<0.1) |
|  | Hypercoagulation | 1 | (<0.1) |
|  | Iron deficiency anemia | 22 | (0.2) |
|  | Microcytic anemia | 1 | (<0.1) |
|  | Thrombocytopenia | 6 | (<0.1) |
|  | Nephrogenic anemia | 2 | (<0.1) |
|  | Heparin-induced thrombocytopenia | 1 | (<0.1) |
|  | Hemorrhagic diathesis | 4 | (<0.1) |
|  | Bone marrow failure | 2 | (<0.1) |
|  | Blood loss anemia | 7 | (<0.1) |
| Immune system disorders | | 1 | (<0.1) |
|  | Amyloidosis senile | 1 | (<0.1) |
| Endocrine disorders | | 4 | (<0.1) |
|  | Adrenal insufficiency | 1 | (<0.1) |
|  | Hypothyroidism | 3 | (<0.1) |
| Metabolism and nutrition disorders | | 173 | (1.6) |
|  | Cachexia | 2 | (<0.1) |
|  | Dehydration | 17 | (0.2) |
|  | Diabetes mellitus | 24 | (0.2) |
|  | Diabetes mellitus inadequate control | 3 | (<0.1) |
|  | Folate deficiency | 1 | (<0.1) |
|  | Gout | 6 | (<0.1) |
|  | Hypercalcemia | 1 | (<0.1) |
|  | Hypercholesterolemia | 9 | (<0.1) |
|  | Hyperglycemia | 3 | (<0.1) |
|  | Hyperkalemia | 23 | (0.2) |
|  | Hyperuricemia | 24 | (0.2) |
|  | Hypoalbuminemia | 1 | (<0.1) |
|  | Hypokalemia | 14 | (0.1) |
|  | Hyponatremia | 9 | (<0.1) |
|  | Marasmus | 19 | (0.2) |
|  | Vitamin B1 deficiency | 1 | (<0.1) |
|  | Vitamin B12 deficiency | 1 | (<0.1) |
|  | Dyslipidemia | 5 | (<0.1) |
|  | Hyperphosphatasemia | 2 | (<0.1) |
|  | Malnutrition | 6 | (<0.1) |
|  | Decreased appetite | 19 | (0.2) |
|  | Hyperlipidemia | 4 | (<0.1) |
|  | Hypophagia | 1 | (<0.1) |
|  | Type 2 diabetes mellitus | 1 | (<0.1) |
| Psychiatric disorders | | 34 | (0.3) |
|  | Aggression | 1 | (<0.1) |
|  | Alcohol problem | 1 | (<0.1) |
|  | Anxiety | 1 | (<0.1) |
|  | Completed suicide | 3 | (<0.1) |
|  | Delirium | 2 | (<0.1) |
|  | Depression | 3 | (<0.1) |
|  | Dysphoria | 1 | (<0.1) |
|  | Eating disorder | 2 | (<0.1) |
|  | Insomnia | 15 | (0.1) |
|  | Irritability | 1 | (<0.1) |
|  | Persecutory delusion | 1 | (<0.1) |
|  | Schizophrenia | 1 | (<0.1) |
|  | Abulia | 1 | (<0.1) |
|  | Affect lability | 1 | (<0.1) |
|  | Mental disorder | 1 | (<0.1) |
| Nervous system disorders | | 462 | (4.3) |
|  | Altered state of consciousness | 3 | (<0.1) |
|  | Aphasia | 2 | (<0.1) |
|  | Brain stem hemorrhage | 5 | (<0.1) |
|  | Carotid artery stenosis | 4 | (<0.1) |
|  | Cerebellar hemorrhage | 3 | (<0.1) |
|  | Cerebral artery embolism | 4 | (<0.1) |
|  | Cerebral hemorrhage | 32 | (0.3) |
|  | Cerebral infarction | 21 | (0.2) |
|  | Cervicobrachial syndrome | 2 | (<0.1) |
|  | Dementia | 9 | (<0.1) |
|  | Dementia Alzheimer's type | 7 | (<0.1) |
|  | Dizziness | 40 | (0.4) |
|  | Dizziness postural | 2 | (<0.1) |
|  | Dysgeusia | 2 | (<0.1) |
|  | Dyskinesia | 1 | (<0.1) |
|  | Embolic stroke | 82 | (0.8) |
|  | Epilepsy | 14 | (0.1) |
|  | Essential tremor | 1 | (<0.1) |
|  | Facial paralysis | 2 | (<0.1) |
|  | Hemorrhage intracranial | 1 | (<0.1) |
|  | Hemorrhagic cerebral infarction | 6 | (<0.1) |
|  | Hemorrhagic stroke | 31 | (0.3) |
|  | Head discomfort | 1 | (<0.1) |
|  | Headache | 19 | (0.2) |
|  | Hypoesthesia | 4 | (<0.1) |
|  | Intracranial aneurysm | 3 | (<0.1) |
|  | Loss of consciousness | 6 | (<0.1) |
|  | Multiple sclerosis | 1 | (<0.1) |
|  | Myasthenia gravis | 1 | (<0.1) |
|  | Myelopathy | 1 | (<0.1) |
|  | Neuralgia | 1 | (<0.1) |
|  | Neuroleptic malignant syndrome | 1 | (<0.1) |
|  | Neuropathy peripheral | 1 | (<0.1) |
|  | Post herpetic neuralgia | 1 | (<0.1) |
|  | Retrograde amnesia | 1 | (<0.1) |
|  | Sciatica | 4 | (<0.1) |
|  | Seizure | 2 | (<0.1) |
|  | Somnolence | 3 | (<0.1) |
|  | Status epilepticus | 2 | (<0.1) |
|  | Subarachnoid hemorrhage | 7 | (<0.1) |
|  | Syncope | 6 | (<0.1) |
|  | Tension headache | 3 | (<0.1) |
|  | Transient ischemic attack | 3 | (<0.1) |
|  | Carotid artery occlusion | 1 | (<0.1) |
|  | Lacunar infarction | 2 | (<0.1) |
|  | Cognitive disorder | 4 | (<0.1) |
|  | Thalamus hemorrhage | 5 | (<0.1) |
|  | Putamen hemorrhage | 2 | (<0.1) |
|  | Embolic cerebral infarction | 3 | (<0.1) |
|  | Ischemic cerebral infarction | 1 | (<0.1) |
|  | Ischemic stroke | 121 | (1.1) |
|  | Partial seizures | 1 | (<0.1) |
|  | Vagus nerve disorder | 1 | (<0.1) |
|  | Parkinson's disease | 4 | (<0.1) |
|  | Vascular encephalopathy | 1 | (<0.1) |
|  | Basal ganglia hemorrhage | 1 | (<0.1) |
|  | Carotid arteriosclerosis | 1 | (<0.1) |
|  | Dementia with Lewy bodies | 2 | (<0.1) |
|  | Postresuscitation encephalopathy | 1 | (<0.1) |
| Eye disorders | | 37 | (0.4) |
|  | Abnormal sensation in eye | 2 | (<0.1) |
|  | Amaurosis fugax | 1 | (<0.1) |
|  | Asthenopia | 1 | (<0.1) |
|  | Cataract | 5 | (<0.1) |
|  | Conjunctival hemorrhage | 14 | (0.1) |
|  | Conjunctivitis allergic | 1 | (<0.1) |
|  | Diabetic retinal edema | 1 | (<0.1) |
|  | Eye pain | 1 | (<0.1) |
|  | Eyelid edema | 2 | (<0.1) |
|  | Ocular hyperemia | 1 | (<0.1) |
|  | Pterygium | 1 | (<0.1) |
|  | Retinal detachment | 1 | (<0.1) |
|  | Retinal hemorrhage | 3 | (<0.1) |
|  | Vision blurred | 1 | (<0.1) |
|  | Visual acuity reduced | 1 | (<0.1) |
|  | Vitreous hemorrhage | 3 | (<0.1) |
|  | Macular hole | 1 | (<0.1) |
|  | Conjunctival hyperemia | 1 | (<0.1) |
| Ear and labyrinth disorders | | 13 | (0.1) |
|  | Ear hemorrhage | 2 | (<0.1) |
|  | Meniere's disease | 1 | (<0.1) |
|  | Tinnitus | 2 | (<0.1) |
|  | Vertigo | 4 | (<0.1) |
|  | Vertigo positional | 2 | (<0.1) |
|  | Sudden hearing loss | 2 | (<0.1) |
| Cardiac disorders | | 412 | (3.9) |
|  | Acute myocardial infarction | 6 | (<0.1) |
|  | Adams–Stokes syndrome | 1 | (<0.1) |
|  | Angina pectoris | 22 | (0.2) |
|  | Angina unstable | 4 | (<0.1) |
|  | Aortic valve incompetence | 1 | (<0.1) |
|  | Aortic valve stenosis | 6 | (<0.1) |
|  | Arrhythmia | 8 | (<0.1) |
|  | Arteriosclerosis coronary artery | 1 | (<0.1) |
|  | Atrial fibrillation | 57 | (0.5) |
|  | Atrial flutter | 17 | (0.2) |
|  | Atrial tachycardia | 2 | (<0.1) |
|  | Atrioventricular block complete | 3 | (<0.1) |
|  | Bradycardia | 12 | (0.1) |
|  | Bundle branch block | 1 | (<0.1) |
|  | Cardiac arrest | 1 | (<0.1) |
|  | Cardiac failure | 117 | (1.1) |
|  | Cardiac failure acute | 34 | (0.3) |
|  | Cardiac failure chronic | 18 | (0.2) |
|  | Cardiac failure congestive | 48 | (0.5) |
|  | Cardiac tamponade | 3 | (<0.1) |
|  | Cardio-respiratory arrest | 3 | (<0.1) |
|  | Cardiogenic shock | 1 | (<0.1) |
|  | Cardiomegaly | 1 | (<0.1) |
|  | Chordae tendinae rupture | 1 | (<0.1) |
|  | Extrasystoles | 1 | (<0.1) |
|  | Left ventricular failure | 4 | (<0.1) |
|  | Mitral valve incompetence | 4 | (<0.1) |
|  | Mitral valve stenosis | 1 | (<0.1) |
|  | Myocardial infarction | 29 | (0.3) |
|  | Myocardial ischemia | 2 | (<0.1) |
|  | Myocardial rupture | 1 | (<0.1) |
|  | Palpitations | 10 | (<0.1) |
|  | Pericardial effusion | 4 | (<0.1) |
|  | Pericardial hemorrhage | 2 | (<0.1) |
|  | Prinzmetal angina | 1 | (<0.1) |
|  | Right ventricular failure | 1 | (<0.1) |
|  | Sinus arrest | 2 | (<0.1) |
|  | Sinus bradycardia | 1 | (<0.1) |
|  | Sinus tachycardia | 1 | (<0.1) |
|  | Supraventricular tachycardia | 1 | (<0.1) |
|  | Tachycardia | 5 | (<0.1) |
|  | Tachycardia paroxysmal | 1 | (<0.1) |
|  | Ventricular fibrillation | 5 | (<0.1) |
|  | Ventricular tachycardia | 3 | (<0.1) |
|  | Atrial thrombosis | 5 | (<0.1) |
|  | Tachyarrhythmia | 2 | (<0.1) |
|  | Cardiopulmonary failure | 1 | (<0.1) |
|  | Acute coronary syndrome | 1 | (<0.1) |
|  | Stress cardiomyopathy | 3 | (<0.1) |
|  | Ventricular dyssynchrony | 1 | (<0.1) |
|  | Tachycardia induced cardiomyopathy | 1 | (<0.1) |
|  | Sinus node dysfunction | 9 | (<0.1) |
|  | Cardiac dysfunction | 1 | (<0.1) |
| Vascular disorders | | 106 | (1.0) |
|  | Aortic aneurysm | 5 | (<0.1) |
|  | Aortic aneurysm rupture | 1 | (<0.1) |
|  | Aortic dissection | 11 | (0.1) |
|  | Blood pressure fluctuation | 1 | (<0.1) |
|  | Circulatory collapse | 1 | (<0.1) |
|  | Flushing | 3 | (<0.1) |
|  | Hematoma | 2 | (<0.1) |
|  | Hemorrhagic infarction | 3 | (<0.1) |
|  | Hypertension | 37 | (0.4) |
|  | Hypotension | 5 | (<0.1) |
|  | Intermittent claudication | 1 | (<0.1) |
|  | Orthostatic hypotension | 2 | (<0.1) |
|  | Peripheral ischemia | 1 | (<0.1) |
|  | Raynaud's phenomenon | 1 | (<0.1) |
|  | Subclavian artery embolism | 1 | (<0.1) |
|  | Thrombosis | 1 | (<0.1) |
|  | Varicose vein | 2 | (<0.1) |
|  | Varicose vein ruptured | 1 | (<0.1) |
|  | Venous thrombosis | 1 | (<0.1) |
|  | Deep vein thrombosis | 2 | (<0.1) |
|  | White coat hypertension | 1 | (<0.1) |
|  | Hemorrhage | 3 | (<0.1) |
|  | Peripheral artery aneurysm | 1 | (<0.1) |
|  | Peripheral artery occlusion | 3 | (<0.1) |
|  | Venous occlusion | 1 | (<0.1) |
|  | Hot flush | 5 | (<0.1) |
|  | Peripheral embolism | 5 | (<0.1) |
|  | Peripheral arterial occlusive disease | 2 | (<0.1) |
|  | Microscopic polyangiitis | 1 | (<0.1) |
|  | Vascular compression | 1 | (<0.1) |
|  | Internal hemorrhage | 5 | (<0.1) |
| Respiratory, thoracic, and mediastinal disorders | | 373 | (3.5) |
|  | Acute pulmonary edema | 1 | (<0.1) |
|  | Acute respiratory distress syndrome | 1 | (<0.1) |
|  | Acute respiratory failure | 5 | (<0.1) |
|  | Asphyxia | 4 | (<0.1) |
|  | Asthma | 14 | (0.1) |
|  | Atelectasis | 2 | (<0.1) |
|  | Bronchitis chronic | 3 | (<0.1) |
|  | Choking | 2 | (<0.1) |
|  | Chronic obstructive pulmonary disease | 7 | (<0.1) |
|  | Cough | 10 | (<0.1) |
|  | Dyspnea | 10 | (<0.1) |
|  | Emphysema | 3 | (<0.1) |
|  | Epistaxis | 144 | (1.3) |
|  | Hemoptysis | 28 | (0.3) |
|  | Hemothorax | 1 | (<0.1) |
|  | Hiccups | 1 | (<0.1) |
|  | Hyperventilation | 1 | (<0.1) |
|  | Interstitial lung disease | 14 | (0.1) |
|  | Lung disorder | 1 | (<0.1) |
|  | Nasal congestion | 1 | (<0.1) |
|  | Pharyngeal hemorrhage | 1 | (<0.1) |
|  | Pleural effusion | 9 | (<0.1) |
|  | Pneumonia aspiration | 54 | (0.5) |
|  | Pneumothorax | 3 | (<0.1) |
|  | Pneumothorax spontaneous | 2 | (<0.1) |
|  | Pulmonary alveolar hemorrhage | 3 | (<0.1) |
|  | Pulmonary congestion | 1 | (<0.1) |
|  | Pulmonary embolism | 1 | (<0.1) |
|  | Pulmonary hemorrhage | 2 | (<0.1) |
|  | Pulmonary hypertension | 1 | (<0.1) |
|  | Respiratory failure | 7 | (<0.1) |
|  | Rhinitis allergic | 7 | (<0.1) |
|  | Rhinorrhea | 2 | (<0.1) |
|  | Sleep apnea syndrome | 1 | (<0.1) |
|  | Throat irritation | 1 | (<0.1) |
|  | Upper respiratory tract inflammation | 31 | (0.3) |
|  | Mediastinal hematoma | 1 | (<0.1) |
|  | Sputum retention | 1 | (<0.1) |
|  | Obstructive airways disorder | 5 | (<0.1) |
|  | Thoracic hemorrhage | 1 | (<0.1) |
|  | Diffuse panbronchiolitis | 1 | (<0.1) |
|  | Pulmonary arterial hypertension | 1 | (<0.1) |
|  | Bronchial hemorrhage | 2 | (<0.1) |
|  | Laryngeal hemorrhage | 1 | (<0.1) |
|  | Laryngeal granuloma | 1 | (<0.1) |
|  | Acute interstitial pneumonitis | 1 | (<0.1) |
|  | Organizing pneumonia | 2 | (<0.1) |
|  | Oropharyngeal discomfort | 1 | (<0.1) |
|  | Oropharyngeal pain | 2 | (<0.1) |
|  | Idiopathic interstitial pneumonia | 1 | (<0.1) |
| Gastrointestinal disorders | | 502 | (4.7) |
|  | Abdominal discomfort | 18 | (0.2) |
|  | Abdominal distension | 3 | (<0.1) |
|  | Abdominal pain | 6 | (<0.1) |
|  | Abdominal pain lower | 1 | (<0.1) |
|  | Abdominal pain upper | 5 | (<0.1) |
|  | Acute abdomen | 1 | (<0.1) |
|  | Anal fissure | 1 | (<0.1) |
|  | Angular cheilitis | 1 | (<0.1) |
|  | Ascites | 1 | (<0.1) |
|  | Chronic gastritis | 9 | (<0.1) |
|  | Colitis ischemic | 4 | (<0.1) |
|  | Colitis ulcerative | 1 | (<0.1) |
|  | Constipation | 26 | (0.2) |
|  | Dental caries | 1 | (<0.1) |
|  | Diarrhea | 17 | (0.2) |
|  | Diverticulum intestinal | 2 | (<0.1) |
|  | Diverticulum intestinal hemorrhagic | 16 | (0.2) |
|  | Duodenal ulcer hemorrhage | 4 | (<0.1) |
|  | Duodenitis | 1 | (<0.1) |
|  | Dyspepsia | 6 | (<0.1) |
|  | Dysphagia | 5 | (<0.1) |
|  | Enterocolitis | 2 | (<0.1) |
|  | Enterocolitis hemorrhagic | 1 | (<0.1) |
|  | Feces discolored | 4 | (<0.1) |
|  | Gastric hemorrhage | 7 | (<0.1) |
|  | Gastric polyps | 2 | (<0.1) |
|  | Gastric ulcer | 8 | (<0.1) |
|  | Gastric ulcer hemorrhage | 19 | (0.2) |
|  | Gastritis | 8 | (<0.1) |
|  | Gastritis erosive | 2 | (<0.1) |
|  | Gastritis hemorrhagic | 3 | (<0.1) |
|  | Gastroesophageal reflux disease | 12 | (0.1) |
|  | Gastrointestinal disorder | 2 | (<0.1) |
|  | Gastrointestinal hemorrhage | 65 | (0.6) |
|  | Gingival bleeding | 55 | (0.5) |
|  | Gingival swelling | 1 | (<0.1) |
|  | Glossitis | 1 | (<0.1) |
|  | Hematemesis | 3 | (<0.1) |
|  | Hematochezia | 16 | (0.2) |
|  | Hemorrhoids | 8 | (<0.1) |
|  | Hiatus hernia | 2 | (<0.1) |
|  | Ileus | 3 | (<0.1) |
|  | Ileus paralytic | 1 | (<0.1) |
|  | Incarcerated inguinal hernia | 1 | (<0.1) |
|  | Inguinal hernia | 8 | (<0.1) |
|  | Intestinal obstruction | 1 | (<0.1) |
|  | Intestinal perforation | 1 | (<0.1) |
|  | Large intestinal ulcer | 1 | (<0.1) |
|  | Large intestine perforation | 1 | (<0.1) |
|  | Melena | 36 | (0.3) |
|  | Mesenteric artery embolism | 2 | (<0.1) |
|  | Mesenteric artery thrombosis | 1 | (<0.1) |
|  | Mouth hemorrhage | 13 | (0.1) |
|  | Nausea | 8 | (<0.1) |
|  | Esophagitis | 1 | (<0.1) |
|  | Oral discomfort | 1 | (<0.1) |
|  | Pancreatic pseudocyst | 1 | (<0.1) |
|  | Pancreatitis | 1 | (<0.1) |
|  | Pancreatitis acute | 4 | (<0.1) |
|  | Periodontal disease | 1 | (<0.1) |
|  | Proctitis | 2 | (<0.1) |
|  | Retroperitoneal hemorrhage | 1 | (<0.1) |
|  | Stomatitis | 5 | (<0.1) |
|  | Upper gastrointestinal hemorrhage | 11 | (0.1) |
|  | Vomiting | 7 | (<0.1) |
|  | Lip hemorrhage | 1 | (<0.1) |
|  | Anal hemorrhage | 9 | (<0.1) |
|  | Tongue hemorrhage | 3 | (<0.1) |
|  | Subileus | 1 | (<0.1) |
|  | Lower gastrointestinal hemorrhage | 12 | (0.1) |
|  | Mechanical ileus | 4 | (<0.1) |
|  | Large intestine polyp | 13 | (0.1) |
|  | Large intestinal hemorrhage | 8 | (<0.1) |
|  | Small intestinal hemorrhage | 3 | (<0.1) |
|  | Epigastric discomfort | 2 | (<0.1) |
|  | Hemorrhoidal hemorrhage | 23 | (0.2) |
|  | Alcoholic pancreatitis | 1 | (<0.1) |
|  | Colitis microscopic | 1 | (<0.1) |
|  | Hypoesthesia oral | 1 | (<0.1) |
|  | Intestinal hemorrhage | 5 | (<0.1) |
|  | Gastrointestinal erosion | 1 | (<0.1) |
|  | Abdominal hernia | 1 | (<0.1) |
|  | Large intestinal ulcer hemorrhage | 2 | (<0.1) |
|  | Tooth socket hemorrhage | 2 | (<0.1) |
|  | Hemorrhagic erosive gastritis | 2 | (<0.1) |
|  | Gastrointestinal polyp hemorrhage | 4 | (<0.1) |
|  | Feces soft | 2 | (<0.1) |
| Hepatobiliary disorders | | 179 | (1.7) |
|  | Alcoholic liver disease | 6 | (<0.1) |
|  | Autoimmune hepatitis | 1 | (<0.1) |
|  | Bile duct stone | 15 | (0.1) |
|  | Cholangitis | 4 | (<0.1) |
|  | Cholangitis acute | 4 | (<0.1) |
|  | Cholecystitis | 11 | (0.1) |
|  | Cholecystitis acute | 12 | (0.1) |
|  | Cholecystitis chronic | 2 | (<0.1) |
|  | Cholelithiasis | 4 | (<0.1) |
|  | Cholestasis | 1 | (<0.1) |
|  | Hepatic congestion | 3 | (<0.1) |
|  | Hepatic cyst | 1 | (<0.1) |
|  | Hepatic failure | 4 | (<0.1) |
|  | Hepatic function abnormal | 91 | (0.9) |
|  | Hepatic steatosis | 3 | (<0.1) |
|  | Hepatitis acute | 1 | (<0.1) |
|  | Hyperbilirubinemia | 1 | (<0.1) |
|  | Jaundice cholestatic | 2 | (<0.1) |
|  | Liver disorder | 19 | (0.2) |
|  | Portal vein thrombosis | 1 | (<0.1) |
|  | Hypertransaminasemia | 1 | (<0.1) |
|  | Drug-induced liver injury | 2 | (<0.1) |
| Skin and subcutaneous tissue disorders | | 206 | (1.9) |
|  | Acute febrile neutrophilic dermatosis | 1 | (<0.1) |
|  | Alopecia | 3 | (<0.1) |
|  | Cold sweat | 1 | (<0.1) |
|  | Decubitus ulcer | 3 | (<0.1) |
|  | Dermatitis | 1 | (<0.1) |
|  | Dermatitis allergic | 1 | (<0.1) |
|  | Dermatitis contact | 1 | (<0.1) |
|  | Drug eruption | 13 | (0.1) |
|  | Ecchymosis | 22 | (0.2) |
|  | Eczema | 6 | (<0.1) |
|  | Eczema asteatotic | 1 | (<0.1) |
|  | Erythema | 7 | (<0.1) |
|  | Hemorrhage subcutaneous | 82 | (0.8) |
|  | Hyperhidrosis | 1 | (<0.1) |
|  | Hyperkeratosis | 1 | (<0.1) |
|  | Palmoplantar keratoderma | 1 | (<0.1) |
|  | Petechiae | 2 | (<0.1) |
|  | Prurigo | 1 | (<0.1) |
|  | Pruritus | 18 | (0.2) |
|  | Purpura | 11 | (0.1) |
|  | Rash | 21 | (0.2) |
|  | Rash generalized | 1 | (<0.1) |
|  | Skin erosion | 1 | (<0.1) |
|  | Skin exfoliation | 1 | (<0.1) |
|  | Swelling face | 1 | (<0.1) |
|  | Urticaria | 3 | (<0.1) |
|  | Xeroderma | 1 | (<0.1) |
|  | Nail bed bleeding | 2 | (<0.1) |
|  | Pruritus generalized | 4 | (<0.1) |
|  | Toxic skin eruption | 1 | (<0.1) |
|  | Hand dermatitis | 1 | (<0.1) |
|  | Skin hemorrhage | 2 | (<0.1) |
| Musculoskeletal and connective tissue disorders | | 94 | (0.9) |
|  | Arthralgia | 4 | (<0.1) |
|  | Arthritis | 1 | (<0.1) |
|  | Back pain | 23 | (0.2) |
|  | Chondrocalcinosis pyrophosphate | 3 | (<0.1) |
|  | Collagen disorder | 1 | (<0.1) |
|  | Hemarthrosis | 4 | (<0.1) |
|  | Lumbar spinal stenosis | 5 | (<0.1) |
|  | Muscle hemorrhage | 3 | (<0.1) |
|  | Muscle spasms | 5 | (<0.1) |
|  | Muscular weakness | 1 | (<0.1) |
|  | Myalgia | 6 | (<0.1) |
|  | Osteoarthritis | 8 | (<0.1) |
|  | Osteoporosis | 5 | (<0.1) |
|  | Pain in extremity | 3 | (<0.1) |
|  | Pathological fracture | 2 | (<0.1) |
|  | Periarthritis | 3 | (<0.1) |
|  | Polymyalgia rheumatica | 1 | (<0.1) |
|  | Polymyositis | 1 | (<0.1) |
|  | Rhabdomyolysis | 2 | (<0.1) |
|  | Rheumatoid arthritis | 1 | (<0.1) |
|  | Rotator cuff syndrome | 4 | (<0.1) |
|  | Spinal osteoarthritis | 2 | (<0.1) |
|  | Tenosynovitis | 2 | (<0.1) |
|  | Mobility decreased | 1 | (<0.1) |
|  | Musculoskeletal stiffness | 5 | (<0.1) |
|  | Inguinal mass | 1 | (<0.1) |
|  | Hematoma muscle | 1 | (<0.1) |
|  | Limb mass | 1 | (<0.1) |
|  | Spinal stenosis | 1 | (<0.1) |
| Renal and urinary disorders | | 276 | (2.6) |
|  | Azotemia | 3 | (<0.1) |
|  | Calculus bladder | 2 | (<0.1) |
|  | Cystitis hemorrhagic | 2 | (<0.1) |
|  | Dysuria | 1 | (<0.1) |
|  | Hematuria | 101 | (1.0) |
|  | Hypertonic bladder | 2 | (<0.1) |
|  | Nephrolithiasis | 3 | (<0.1) |
|  | Nephropathy toxic | 1 | (<0.1) |
|  | Nephrosclerosis | 1 | (<0.1) |
|  | Neurogenic bladder | 4 | (<0.1) |
|  | Pollakiuria | 6 | (<0.1) |
|  | Renal artery stenosis | 1 | (<0.1) |
|  | Renal disorder | 20 | (0.2) |
|  | Renal failure | 7 | (<0.1) |
|  | Renal hemorrhage | 1 | (<0.1) |
|  | Urinary bladder hemorrhage | 8 | (<0.1) |
|  | Urinary incontinence | 1 | (<0.1) |
|  | Urinary retention | 2 | (<0.1) |
|  | Tubulointerstitial nephritis | 1 | (<0.1) |
|  | Urethral hemorrhage | 1 | (<0.1) |
|  | Hemorrhage urinary tract | 7 | (<0.1) |
|  | Urethral meatus stenosis | 1 | (<0.1) |
|  | Renal impairment | 86 | (0.8) |
|  | Chronic kidney disease | 9 | (<0.1) |
|  | Urethral stenosis | 1 | (<0.1) |
|  | Ureteric hemorrhage | 1 | (<0.1) |
|  | Acute kidney injury | 9 | (<0.1) |
|  | End stage renal disease | 2 | (<0.1) |
|  | Ureterolithiasis | 4 | (<0.1) |
| Reproductive system and breast disorders | | 29 | (0.3) |
|  | Benign prostatic hyperplasia | 5 | (<0.1) |
|  | Gynecomastia | 3 | (<0.1) |
|  | Hematospermia | 1 | (<0.1) |
|  | Menorrhagia | 3 | (<0.1) |
|  | Metrorrhagia | 6 | (<0.1) |
|  | Prostatic hemorrhage | 1 | (<0.1) |
|  | Prostatitis | 3 | (<0.1) |
|  | Uterine hemorrhage | 2 | (<0.1) |
|  | Genital erythema | 1 | (<0.1) |
|  | Genital hemorrhage | 4 | (<0.1) |
| General disorders and administration site conditions | | 130 | (1.2) |
|  | Asthenia | 3 | (<0.1) |
|  | Chest discomfort | 2 | (<0.1) |
|  | Chest pain | 2 | (<0.1) |
|  | Death | 23 | (0.2) |
|  | Drowning | 2 | (<0.1) |
|  | Face edema | 1 | (<0.1) |
|  | Feeling abnormal | 3 | (<0.1) |
|  | Feeling hot | 1 | (<0.1) |
|  | Injection site bruising | 1 | (<0.1) |
|  | Injection site hemorrhage | 2 | (<0.1) |
|  | Malaise | 5 | (<0.1) |
|  | Edema | 8 | (<0.1) |
|  | Edema peripheral | 25 | (0.2) |
|  | Pain | 3 | (<0.1) |
|  | Pyrexia | 3 | (<0.1) |
|  | Sudden death | 11 | (0.1) |
|  | Thirst | 3 | (<0.1) |
|  | Terminal state | 1 | (<0.1) |
|  | Sudden cardiac death | 6 | (<0.1) |
|  | General physical health deterioration | 7 | (<0.1) |
|  | Edema due to cardiac disease | 2 | (<0.1) |
|  | Cardiac death | 1 | (<0.1) |
|  | Puncture site hemorrhage | 1 | (<0.1) |
|  | Implant site hemorrhage | 1 | (<0.1) |
|  | Vessel puncture site hemorrhage | 1 | (<0.1) |
|  | Adverse event | 1 | (<0.1) |
|  | Unevaluable event | 1 | (<0.1) |
|  | Implant site hematoma | 3 | (<0.1) |
|  | Stent-graft endoleak | 2 | (<0.1) |
|  | Disuse syndrome | 1 | (<0.1) |
|  | Vaccination site bruising | 1 | (<0.1) |
|  | Medical device site hemorrhage | 1 | (<0.1) |
|  | Multiple organ dysfunction syndrome | 4 | (<0.1) |
|  | Therapeutic product effect incomplete | 1 | (<0.1) |
| Investigations | | 275 | (2.6) |
|  | Activated partial thromboplastin time prolonged | 21 | (0.2) |
|  | Alanine aminotransferase increased | 11 | (0.1) |
|  | Amylase increased | 1 | (<0.1) |
|  | Aspartate aminotransferase increased | 8 | (<0.1) |
|  | Bleeding time prolonged | 3 | (<0.1) |
|  | Blood bilirubin increased | 9 | (<0.1) |
|  | Blood cholesterol increased | 6 | (<0.1) |
|  | Blood creatine phosphokinase increased | 3 | (<0.1) |
|  | Blood creatinine abnormal | 3 | (<0.1) |
|  | Blood creatinine decreased | 1 | (<0.1) |
|  | Blood creatinine increased | 28 | (0.3) |
|  | Blood fibrinogen decreased | 1 | (<0.1) |
|  | Blood iron decreased | 1 | (<0.1) |
|  | Blood potassium decreased | 1 | (<0.1) |
|  | Blood potassium increased | 2 | (<0.1) |
|  | Blood pressure decreased | 2 | (<0.1) |
|  | Blood pressure increased | 23 | (0.2) |
|  | Blood pressure systolic increased | 2 | (<0.1) |
|  | Blood sodium increased | 1 | (<0.1) |
|  | Blood triglycerides increased | 3 | (<0.1) |
|  | Blood urea increased | 13 | (0.1) |
|  | Blood uric acid increased | 2 | (<0.1) |
|  | C-reactive protein increased | 19 | (0.2) |
|  | Creatinine renal clearance decreased | 6 | (<0.1) |
|  | Electrocardiogram abnormal | 1 | (<0.1) |
|  | Fibrin D dimer increased | 5 | (<0.1) |
|  | Fibrin degradation products increased | 3 | (<0.1) |
|  | Gamma-glutamyltransferase increased | 34 | (0.3) |
|  | Glomerular filtration rate decreased | 1 | (<0.1) |
|  | Glycosylated hemoglobin increased | 2 | (<0.1) |
|  | Blood urine present | 3 | (<0.1) |
|  | Hemoglobin decreased | 15 | (0.1) |
|  | Heart rate decreased | 1 | (<0.1) |
|  | International normalized ratio abnormal | 1 | (<0.1) |
|  | International normalized ratio increased | 40 | (0.4) |
|  | Liver function test abnormal | 3 | (<0.1) |
|  | Low density lipoprotein increased | 3 | (<0.1) |
|  | Platelet count decreased | 110 | (0.1) |
|  | Prothrombin level decreased | 1 | (<0.1) |
|  | Prothrombin time abnormal | 1 | (<0.1) |
|  | Prothrombin time prolonged | 13 | (0.1) |
|  | Weight decreased | 2 | (<0.1) |
|  | Weight increased | 1 | (<0.1) |
|  | White blood cell count decreased | 2 | (<0.1) |
|  | Carbohydrate antigen 19-9 increased | 1 | (<0.1) |
|  | Platelet count increased | 1 | (<0.1) |
|  | Blood bilirubin abnormal | 1 | (<0.1) |
|  | Blood alkaline phosphatase increased | 11 | (0.1) |
|  | Blood alkaline phosphatase abnormal | 1 | (<0.1) |
|  | Urine output decreased | 1 | (<0.1) |
|  | Hepatic enzyme increased | 4 | (<0.1) |
|  | Renal function test abnormal | 2 | (<0.1) |
|  | Blood uric acid abnormal | 1 | (<0.1) |
|  | Occult blood | 2 | (<0.1) |
|  | Occult blood positive | 17 | (0.2) |
|  | Hepatic enzyme abnormal | 1 | (<0.1) |
|  | Cell marker increased | 2 | (<0.1) |
|  | C-reactive protein abnormal | 1 | (<0.1) |
|  | Helicobacter test positive | 1 | (<0.1) |
|  | Transvalvular pressure gradient | 1 | (<0.1) |
|  | Liver function test increased | 4 | (<0.1) |
| Injury, poisoning, and procedural complications | | 162 | (1.5) |
|  | Arthropod sting | 1 | (<0.1) |
|  | Brain herniation | 1 | (<0.1) |
|  | Chilblains | 1 | (<0.1) |
|  | Clavicle fracture | 2 | (<0.1) |
|  | Extradural hematoma | 2 | (<0.1) |
|  | Fall | 28 | (0.3) |
|  | Femoral neck fracture | 8 | (<0.1) |
|  | Femur fracture | 10 | (<0.1) |
|  | Foot fracture | 2 | (<0.1) |
|  | Fracture | 2 | (<0.1) |
|  | Fractured ischium | 1 | (<0.1) |
|  | Fractured sacrum | 2 | (<0.1) |
|  | Head injury | 1 | (<0.1) |
|  | Humerus fracture | 6 | (<0.1) |
|  | Incisional hernia | 1 | (<0.1) |
|  | Injury | 1 | (<0.1) |
|  | Joint dislocation | 1 | (<0.1) |
|  | Ligament sprain | 1 | (<0.1) |
|  | Patella fracture | 3 | (<0.1) |
|  | Pneumoconiosis | 1 | (<0.1) |
|  | Radius fracture | 2 | (<0.1) |
|  | Rib fracture | 5 | (<0.1) |
|  | Road traffic accident | 3 | (<0.1) |
|  | Spinal compression fracture | 25 | (0.2) |
|  | Subcutaneous hematoma | 8 | (<0.1) |
|  | Subdural hematoma | 27 | (0.3) |
|  | Subdural hemorrhage | 2 | (<0.1) |
|  | Traumatic hematoma | 1 | (<0.1) |
|  | Ulna fracture | 2 | (<0.1) |
|  | Traumatic fracture | 2 | (<0.1) |
|  | Cervical vertebral fracture | 1 | (<0.1) |
|  | Contusion | 12 | (0.1) |
|  | Post procedural hemorrhage | 4 | (<0.1) |
|  | Wound | 1 | (<0.1) |
|  | Traumatic hemorrhage | 4 | (<0.1) |
|  | Anastomotic hemorrhage | 1 | (<0.1) |
|  | Near drowning | 1 | (<0.1) |
|  | Tongue injury | 1 | (<0.1) |
|  | Upper limb fracture | 2 | (<0.1) |
|  | Lower limb fracture | 1 | (<0.1) |
|  | Tracheal hemorrhage | 1 | (<0.1) |
|  | Bone contusion | 1 | (<0.1) |
|  | Heat illness | 9 | (<0.1) |
|  | Spinal column injury | 1 | (<0.1) |
|  | Procedural hemorrhage | 2 | (<0.1) |
|  | Skin wound | 1 | (<0.1) |
|  | Radiation associated hemorrhage | 1 | (<0.1) |
|  | Traumatic hemothorax | 2 | (<0.1) |
|  | Incomplete spinal fusion | 1 | (<0.1) |
| Surgical and medical procedures | | 10 | (<0.1) |
|  | Mitral valve replacement | 1 | (<0.1) |
|  | Gastrostomy | 1 | (<0.1) |
|  | Cardiac pacemaker replacement | 4 | (<0.1) |
|  | Hospitalization | 1 | (<0.1) |
|  | Joint arthroplasty | 1 | (<0.1) |
|  | Cataract operation | 1 | (<0.1) |
|  | Cancer surgery | 1 | (<0.1) |
| Product issues | | 2 | (<0.1) |
|  | Device battery issue | 2 | (<0.1) |

Multiple adverse events were reported in some patients. Classification is based on system organ class in the Medical Dictionary for Regulatory Activities (MedDRA), version 20.0.
