# Supplementary material for: Real-world safety and effectiveness of rivaroxaban using Japan-specific dosage during long-term follow-up in patients with atrial fibrillation: XAPASS
Source: PLoS One. 2021 Jun 11;16(6):e0251325. doi: 10.1371/journal.pone.0251325 (PMC8195353; doi:10.1371/journal.pone.0251325)
Supplement: S4 Table — (DOCX) [file pone.0251325.s005.docx]

**S4 Table.** **Incidences of effectiveness outcomes in patient subgroups.**

| **Patient population** | **N** | **Stroke/non-CNS SE** | | **Ischemic stroke** | | **Stroke/non-CNS SE/MI** | |
| --- | --- | --- | --- | --- | --- | --- | --- |
|  |  | **Crude incidence, n** | **Incidence, events per 100 patient-years** | **Crude incidence, n** | **Incidence, events per 100 patient-years** | **Crude incidence, n** | **Incidence, events per 100 patient-years** |
| Overall | 10,628 | 316 | 1.19 | 227 | 0.86 | 350 | 1.32 |
| Age, years |  |  |  |  |  |  |  |
| <65 | 1901 | 27 | 0.55 | 16 | 0.33 | 30 | 0.61 |
| <75 | 5450 | 112 | 0.78 | 74 | 0.52 | 120 | 0.84 |
| ≥65 | 5178 | 289 | 1.34 | 211 | 0.97 | 320 | 1.48 |
| ≥75 | 8727 | 204 | 1.68 | 153 | 1.26 | 230 | 1.89 |
| ≥85 | 1136 | 44 | 2.13 | 31 | 1.50 | 56 | 2.72 |
| Sex |  |  |  |  |  |  |  |
| Male | 6575 | 197 | 1.20 | 136 | 0.83 | 217 | 1.32 |
| Female | 4053 | 119 | 1.18 | 91 | 0.90 | 133 | 1.32 |
| Body weight, kg |  |  |  |  |  |  |  |
| ≤50 | 2051 | 74 | 1.66 | 50 | 1.12 | 85 | 1.91 |
| >50 | 7837 | 223 | 1.10 | 164 | 0.80 | 245 | 1.20 |
| BMI, kg/m^2^ |  |  |  |  |  |  |  |
| <18.5 | 587 | 18 | 1.56 | 12 | 1.04 | 22 | 1.91 |
| 18.5 to <25 | 4913 | 156 | 1.27 | 110 | 0.89 | 172 | 1.40 |
| 25 to <30 | 2396 | 66 | 1.04 | 46 | 0.72 | 72 | 1.14 |
| ≥30 | 549 | 13 | 0.86 | 11 | 0.72 | 15 | 0.99 |
| Creatinine clearance, mL/min |  |  |  |  |  |  |  |
| <15 | 3 | 0 | 0 | 0 | 0 | 0 | 0 |
| 15 to <30 | 292 | 10 | 2.15 | 6 | 1.29 | 15 | 3.23 |
| 30 to <50 | 2228 | 93 | 1.88 | 67 | 1.35 | 103 | 2.08 |
| 50 to <80 | 4551 | 140 | 1.18 | 105 | 0.88 | 153 | 1.29 |
| ≥80 | 2718 | 51 | 0.70 | 34 | 0.47 | 56 | 0.77 |
| CHADS_2_ score |  |  |  |  |  |  |  |
| 0 | 895 | 10 | 0.46 | 4 | 0.18 | 11 | 0.50 |
| 1 | 2599 | 38 | 0.55 | 30 | 0.43 | 42 | 0.61 |
| 2 | 3202 | 92 | 1.10 | 63 | 0.75 | 100 | 1.19 |
| 3 | 2085 | 79 | 1.59 | 51 | 1.02 | 88 | 1.77 |
| 4 | 1259 | 63 | 2.25 | 48 | 1.71 | 71 | 2.54 |
| 5 | 484 | 26 | 2.60 | 24 | 2.40 | 29 | 2.91 |
| 6 | 104 | 8 | 3.38 | 7 | 2.9475 | 9 | 3.80 |
| CHA_2_DS_2_-VASc score |  |  |  |  |  |  |  |
| 0 | 272 | 2 | 0.33 | 1 | 0.16 | 3 | 0.49 |
| 1 | 999 | 14 | 0.54 | 9 | 0.35 | 15 | 0.58 |
| 2 | 1805 | 20 | 0.42 | 13 | 0.27 | 22 | 0.46 |
| 3 | 2488 | 74 | 1.13 | 49 | 0.75 | 76 | 1.16 |
| 4 | 2365 | 72 | 1.20 | 49 | 0.81 | 83 | 1.38 |
| 5 | 1562 | 77 | 2.14 | 57 | 1.58 | 84 | 2.33 |
| 6 | 782 | 36 | 2.10 | 31 | 1.80 | 44 | 2.57 |
| 7 | 298 | 17 | 2.95 | 14 | 2.43 | 19 | 3.30 |
| 8 | 54 | 4 | 3.62 | 4 | 3.62 | 4 | 3.62 |
| 9 | 3 | 0 | 0 | 0 | 0 | 0 | 0 |
| Modified HAS-BLED score^a^ |  |  |  |  |  |  |  |
| 0 | 1354 | 11 | 0.31 | 7 | 0.20 | 13 | 0.37 |
| 1 | 4508 | 92 | 0.78 | 61 | 0.51 | 104 | 0.88 |
| 2 | 3258 | 127 | 1.62 | 96 | 1.22 | 138 | 1.76 |
| 3 | 1227 | 68 | 2.59 | 50 | 1.90 | 74 | 2.81 |
| 4 | 250 | 14 | 2.52 | 11 | 1.96 | 17 | 3.07 |
| 5 | 29 | 4 | 8.94 | 2 | 4.46 | 4 | 8.94 |
| 6 | 1 | 0 | 0 | 0 | 0 | 0 | 0 |
| 7 | 0 | – | – | – | – | – | – |
| 8 | 0 | – | – | – | – | – | – |
| Baseline comorbidities | | | | | | | |
| Congestive heart failure | 2671 | 97 | 1.55 | 76 | 1.21 | 106 | 1.69 |
| Hypertension | 7982 | 258 | 1.27 | 185 | 0.91 | 288 | 1.42 |
| Diabetes mellitus | 2427 | 82 | 1.33 | 59 | 0.95 | 91 | 1.48 |
| Prior ischemic stroke/TIA | 2441 | 120 | 2.20 | 94 | 1.72 | 132 | 2.42 |
| Vascular disease^b^ | 426 | 19 | 1.94 | 14 | 1.43 | 26 | 2.67 |
| Hepatic dysfunction | 720651 | 32 | 1.75 | 25 | 1.36 | 34 | 1.86 |
| Type of AF |  |  |  |  |  |  |  |
| Paroxysmal | 3589 | 87 | 1.00 | 57 | 0.65 | 99 | 1.14 |
| Persistent | 3821 | 112 | 1.19 | 76 | 0.80 | 125 | 1.32 |
| Permanent | 2605 | 98 | 1.41 | 81 | 1.16 | 106 | 1.53 |
| Oral antiplatelet use | 469 | 28 | 2.73 | 19 | 1.85 | 32 | 3.12 |

^a^ Maximum score is 8 because the labile international normalized ratio was excluded.

^b^ Vascular disease is defined as myocardial infarction and/or peripheral artery disease and/or aortic plaque.

Abbreviations: AF, atrial fibrillation; BMI, body mass index; CHADS_2_, Congestive heart failure, Hypertension, Age ≥75 years, Diabetes mellitus, previous Stroke/TIA (2 points); CHA_2_DS_2_-VASc, Congestive heart failure, Hypertension, Age (65–74 years, 1 point; ≥75 years, 2 points), Diabetes mellitus, previous Stroke/TIA (2 points), Vascular disease and female sex; Modified HAS-BLED, Hypertension, Abnormal renal or liver function, previous Stroke, previous major or predisposition to Bleeding, Labile international normalized ratio (excluded from this analysis), Elderly (>65 years), medication use predisposing to bleeding, and previous Drug or alcohol use; TIA, transient ischemic attack.
