# Supplementary material for: Real-world safety and effectiveness of rivaroxaban using Japan-specific dosage during long-term follow-up in patients with atrial fibrillation: XAPASS
Source: PLoS One. 2021 Jun 11;16(6):e0251325. doi: 10.1371/journal.pone.0251325 (PMC8195353; doi:10.1371/journal.pone.0251325)
Supplement: S5 Table — (DOCX) [file pone.0251325.s006.docx]

**S5 Table.** **Characteristics of patients in the safety analysis population who started rivaroxaban treatment at the recommended dose.**

| **Characteristic** | **All patients (****N = 6953)^a^** | |
| --- | --- | --- |
| Age, years, mean (SD) | 72.3 (10.3) | |
| ≥65 | 5477 | 78.8 |
| ≥75 | 3075 | 44.2 |
| ≥85 | 790 | 11.4 |
| Female sex | 2533 | 36.4 |
| Height, cm, mean (SD) | 160.6 (9.98) | |
| Body weight, kg, mean (SD) | 61.5 (13.45) | |
| ≤50 | 1467 | 21.1 |
| >50 | 5483 | 78.9 |
| Unknown | 3 | 0.04 |
| BMI, kg/m^2^, mean (SD) | 23.8 (4.02) | |
| <18.5 | 427 | 6.1 |
| 18.5 to <25 | 3554 | 51.1 |
| 25 to <30 | 1628 | 23.4 |
| ≥30 | 365 | 5.3 |
| Unknown | 979 | 14.1 |
| Serum creatinine, mg/dL, mean (SD) | 0.87 (0.26) | |
| Unknown | 0 | – |
| Creatinine clearance, mL/min, mean (SD) | 68.2 (28.7) | |
| <15 | 3 | 0.04 |
| 15 to <30 | 276 | 4.0 |
| 30 to <50 | 1973 | 28.4 |
| 50 to <80 | 2523 | 36.3 |
| ≥80 | 2178 | 31.3 |
| Unknown | 0 | – |
| CHADS_2_ score, mean (SD) | 2.2 (1.3) | |
| 0 | 617 | 8.9 |
| 1 | 1742 | 25.1 |
| 2 | 2057 | 29.6 |
| 3 | 1362 | 19.6 |
| 4 | 798 | 11.5 |
| 5 | 313 | 4.5 |
| 6 | 64 | 0.9 |
| CHA_2_DS_2_-VASc score, mean (SD) | 3.4 (1.7) | |
| 0 | 206 | 3.0 |
| 1 | 747 | 10.7 |
| 2 | 1260 | 18.1 |
| 3 | 1527 | 22.0 |
| 4 | 1497 | 21.5 |
| 5 | 1009 | 14.5 |
| 6 | 475 | 6.8 |
| 7 | 194 | 2.8 |
| 8 | 37 | 0.5 |
| 9 | 1 | 0.01 |
| Modified HAS-BLED score, mean (SD)^b^ | 1.5 (1.0) | |
| 0 | 1049 | 15.1 |
| 1 | 2822 | 40.6 |
| 2 | 2082 | 30.0 |
| 3 | 817 | 11.8 |
| 4 | 160 | 2.3 |
| 5 | 22 | 0.3 |
| 6 | 0 | – |
| 7 | 0 | – |
| 8 | 0 | – |
| Baseline comorbidities |  |  |
| Congestive heart failure | 1792 | 25.8 |
| Hypertension | 5238 | 75.3 |
| Diabetes mellitus | 1614 | 23.2 |
| Prior ischemic stroke/TIA | 1623 | 23.3 |
| Vascular disease^c^ | 275 | 4.0 |
| Hepatic dysfunction | 467 | 6.7 |
| Type of AF |  |  |
| Paroxysmal | 2297 | 33.0 |
| Persistent | 2515 | 36.2 |
| Permanent | 1734 | 24.9 |
| Other | 19 | 0.3 |
| Unknown | 388 | 5.6 |
| Oral antiplatelet use | 323 | 4.7 |

^a^ Data are presented as number and proportion of patients unless otherwise stated.

^b^ Maximum score is 8 because the labile international normalized ratio was excluded.

^c^ Vascular disease is defined as myocardial infarction and/or peripheral artery disease and/or aortic plaque.

Abbreviations: AF, atrial fibrillation; BMI, body mass index; CHADS_2_, Congestive heart failure, Hypertension, Age ≥75 years, Diabetes mellitus, previous Stroke/TIA (2 points); CHA_2_DS_2_-VASc, Congestive heart failure, Hypertension, Age (65–74 years, 1 point; ≥75 years, 2 points), Diabetes mellitus, previous Stroke/TIA (2 points), Vascular disease and female sex; Modified HAS-BLED, Hypertension, Abnormal renal or liver function, previous Stroke, previous major or predisposition to Bleeding, Labile international normalized ratio (excluded from this analysis), Elderly (>65 years), medication use predisposing to bleeding, and previous Drug or alcohol use; SD, standard deviation; TIA, transient ischemic attack.
