# Supplementary material for: Real-world safety and effectiveness of rivaroxaban using Japan-specific dosage during long-term follow-up in patients with atrial fibrillation: XAPASS
Source: PLoS One. 2021 Jun 11;16(6):e0251325. doi: 10.1371/journal.pone.0251325 (PMC8195353; doi:10.1371/journal.pone.0251325)
Supplement: S6 Table — (DOCX) [file pone.0251325.s007.docx]

**S6 Table.** **Safety and effectiveness outcomes in patients who started rivaroxaban treatment at the recommended dose.**

|  | **Crude incidence, n (%)** | **Incidence, events per 100 patient-years (95% CI)** |
| --- | --- | --- |
| **Safety outcomes (N = 6953)** |  |  |
| Any bleeding | 658 (9.5) | 4.00 (3.69–4.30) |
| Major bleeding | 203 (2.9) | 1.18 (1.02–1.34) |
| Fatal bleeding | 21 (0.3) | 0.12 (0.07–0.17) |
| Critical organ bleeding | 91 (1.3) | 0.53 (0.42–0.63) |
| Intracranial hemorrhage | 84 (1.2) | 0.48 (0.38–0.59) |
| Hemoglobin decrease ≥2 g/dL | 78 (1.1) | 0.45 (0.35–0.55) |
| Transfusion of ≥2 units of packed red blood cells or whole blood | 27 (0.4) | 0.16 (0.10–0.21) |
| Non-major bleeding | 473 (6.8) | 2.84 (2.59–3.10) |
| All-cause mortality | 295 (4.2) | 1.70 (1.50–1.89) |
| **Effectiveness outcomes (N = 6930)** |  |  |
| Stroke/non-CNS SE/MI | 229 (3.3) | 1.33 (1.16–1.51) |
| Stroke | 200 (2.9) | 1.16 (1.00–1.33) |
| Ischemic stroke | 150 (2.2) | 0.87 (0.73–1.01) |
| Hemorrhagic stroke | 59 (0.9) | 0.34 (0.25–0.43) |
| Non-CNS SE | 7 (0.1) | 0.04 (0.01–0.07) |
| MI | 24 (0.4) | 0.14 (0.08–0.19) |
| Stroke/non-CNS SE | 206 (3.0) | 1.20 (1.04–1.36) |

Abbreviations: CI, confidence interval; CNS, central nervous system; MI, myocardial infarction; SE, systemic embolism.
