# Supplementary figures and images for: Real-world safety and effectiveness of rivaroxaban using Japan-specific dosage during long-term follow-up in patients with atrial fibrillation: XAPASS
Source: PLoS One. 2021 Jun 11;16(6):e0251325. doi: 10.1371/journal.pone.0251325 (PMC8195353; doi:10.1371/journal.pone.0251325)

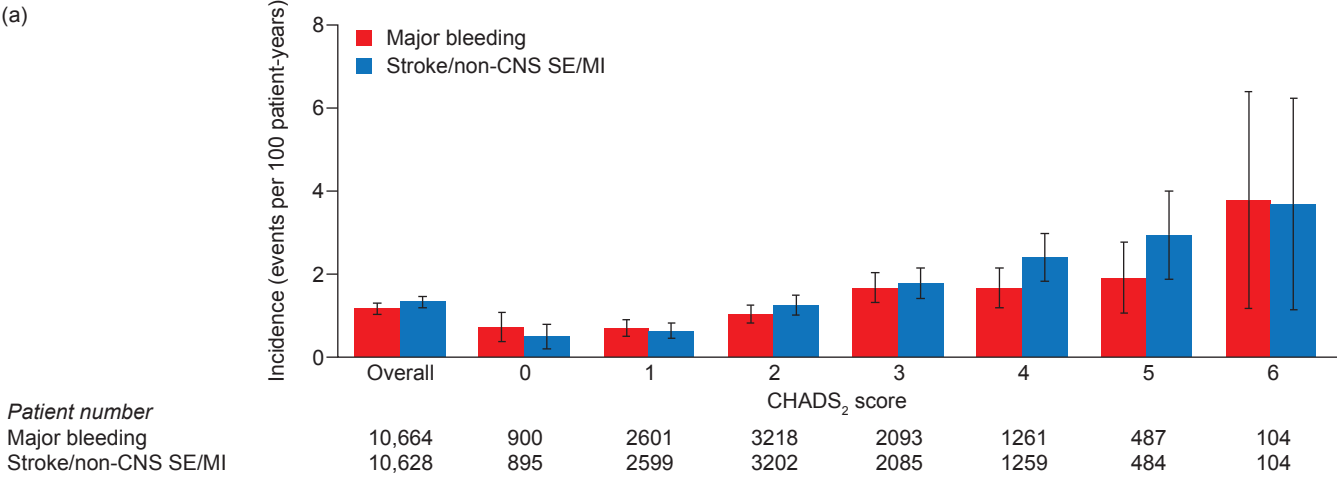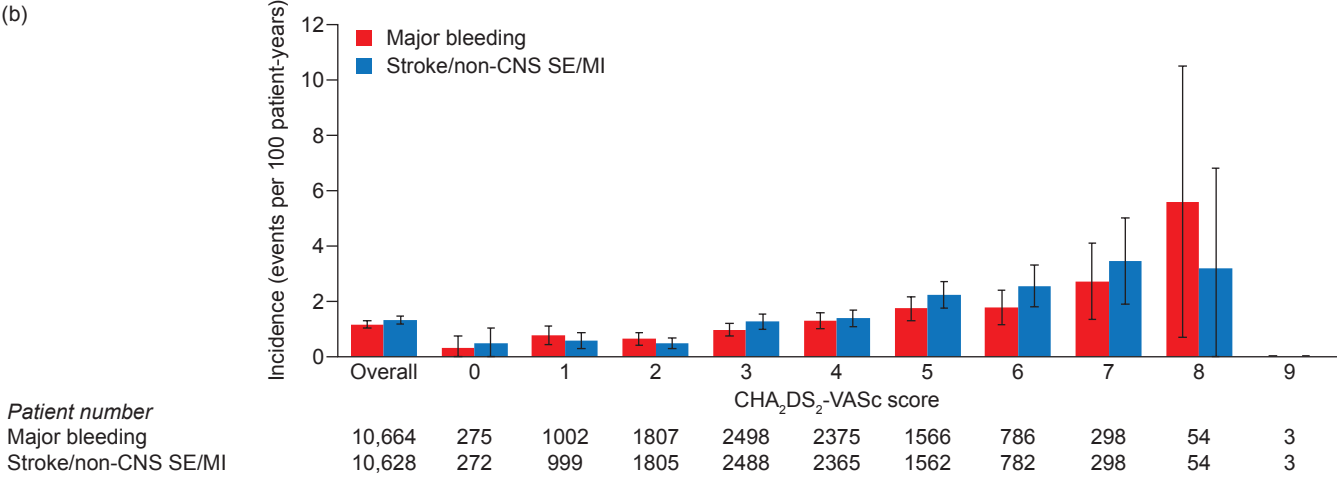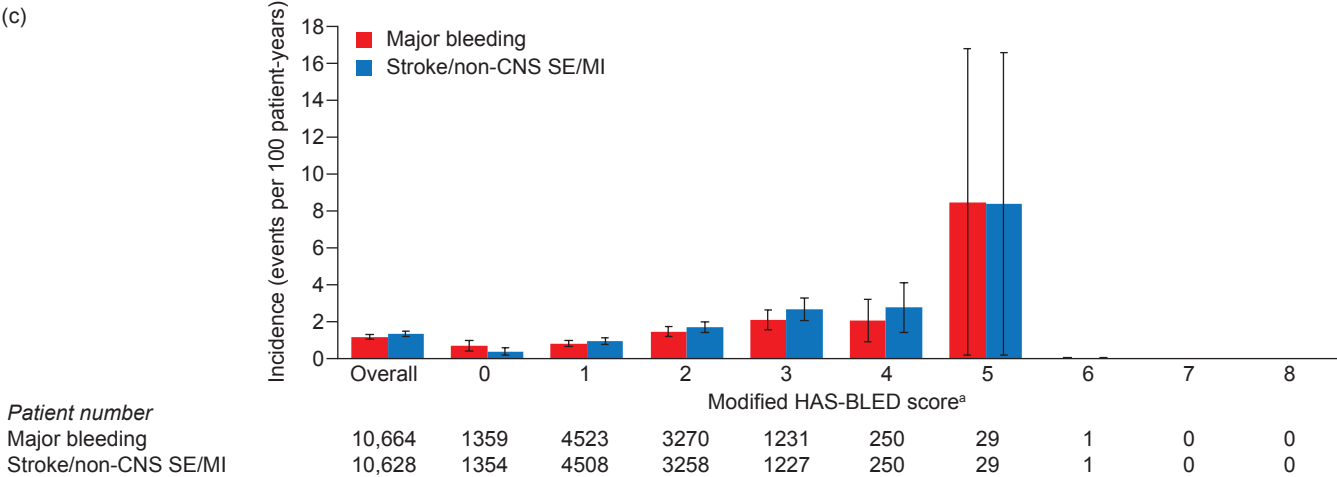

Supplement: S1 Fig — The incidences of major bleeding and the primary composite effectiveness outcome of stroke, non-CNS SE and MI by baseline CHADS2 score (a), CHA2DS2-VASc score (b), and modified HAS-BLED score (c). (PDF) [file pone.0251325.s008.pdf]

(a)

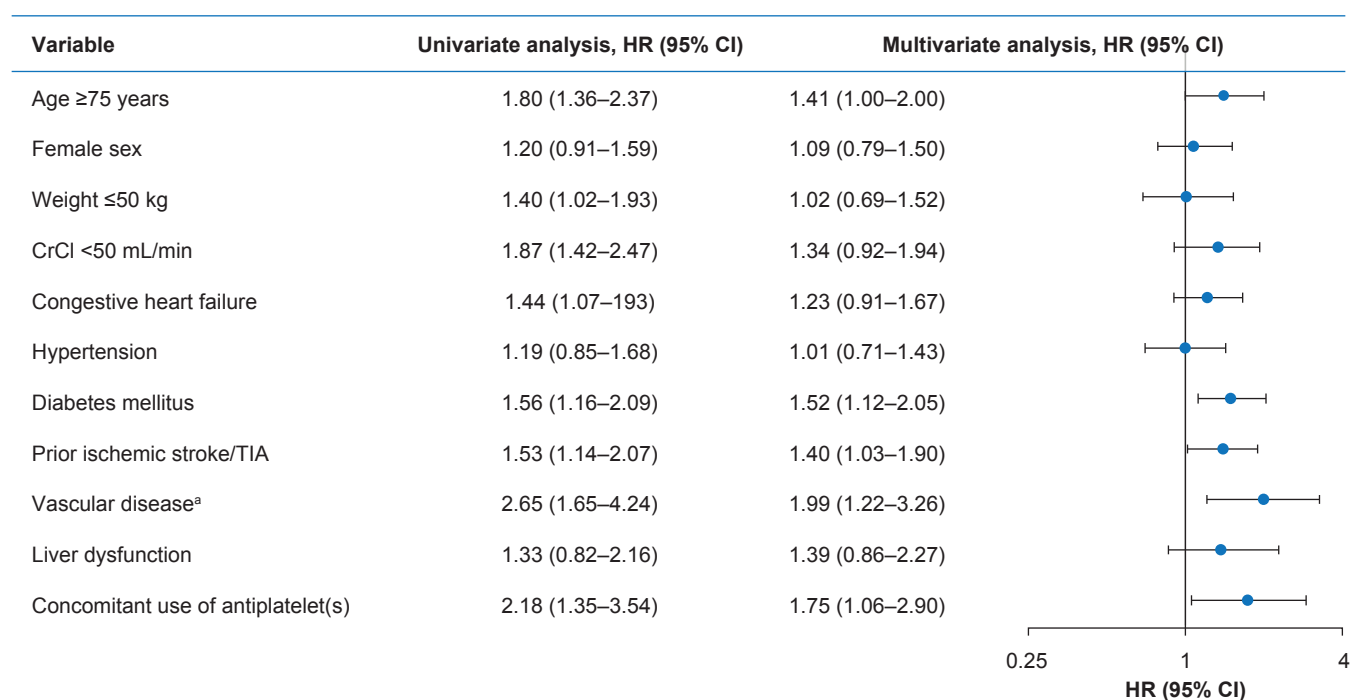

(b)

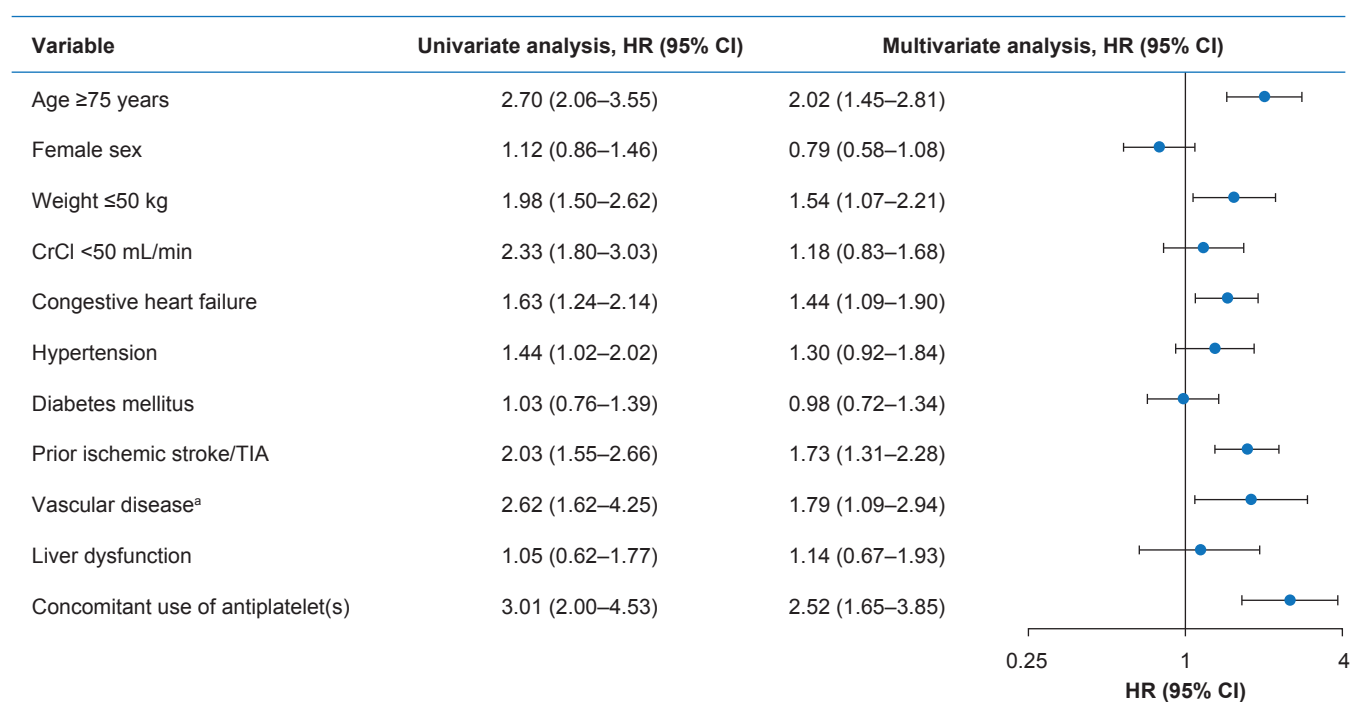

Supplement: S2 Fig — Univariate and multivariate Cox regression analyses for major bleeding (a) and primary composite effectiveness outcome of stroke, non-central nervous system systemic embolism, and myocardial infarction (b) in patients who started rivaroxaban treatment at the recommended dose. (PDF) [file pone.0251325.s009.pdf]
