# Supplementary material for: Real-world safety and effectiveness of rivaroxaban using Japan-specific dosage during long-term follow-up in patients with atrial fibrillation: XAPASS
Source: PLoS One. 2021 Jun 11;16(6):e0251325. doi: 10.1371/journal.pone.0251325 (PMC8195353; doi:10.1371/journal.pone.0251325)
Supplement: S1 Appendix — (DOCX) [file pone.0251325.s010.docx]

**S1 Appendix. Details of the steering committee members for the study.**

| **Name** | **Affiliation** |
| --- | --- |
| Satoshi Ogawa | International University of Health & Welfare Mita Hospital, Tokyo, Japan |
| Takanori Ikeda | Department of Cardiovascular Medicine, Toho University Graduate School of Medicine, Tokyo, Japan |
| Takanari Kitazono | Department of Medicine and Clinical Science, Graduate School of Medical Sciences, Kyushu University, Fukuoka, Japan |
| Jyoji Nakagawara | Osaka Namba Clinic, Osaka, Japan |
| Kazuo Minematsu | National Cerebral and Cardiovascular Center, Suita, Japan |
| Susumu Miyamoto | Department of Neurosurgery, Kyoto University Graduate School of Medicine, Kyoto, Japan |
| Yuji Murakawa | The 4th Department of Internal Medicine, Teikyo University School of Medicine, Mizonokuchi Hospital, Kawasaki, Japan |
