# Supplementary material for: Real-world safety and effectiveness of rivaroxaban using Japan-specific dosage during long-term follow-up in patients with atrial fibrillation: XAPASS
Source: PLoS One. 2021 Jun 11;16(6):e0251325. doi: 10.1371/journal.pone.0251325 (PMC8195353; doi:10.1371/journal.pone.0251325)
Supplement: S1 File — (PDF) [file pone.0251325.s011.pdf]

Post-Marketing Surveillance Protocol

Implementation Guidelines for Special Drug Use Investigation of Xarelto

(Stroke Prevention in Atrial Fibrillation)

-Prevention of ischemic stroke and systemic embolism in patients with non-valvulae atrial fibrillation-

Bayer Yakuhin Ltd.

Prepared on January 20, 2012

Table of Contents

1. OBJECTIVES OF INVESTIGATION
2. PLANNED NUMBER OF PATIENTS
3. TARGET POPULATION AND DOSAGE REGIMEN
4. STUDY METHODS
5. PLANNED STUDY PERIOD
6. STUDY VARIABLES
7. ESTABLISHMENT OF THE POST-MARKETING SURVEILLANCE COMMITTEE
8. CONTACT FOR INQUIRIES

1. OBJECTIVES OF INVESTIGATION

This investigation will be conducted in accordance with the regulation in Article 14-4 (Re-Examination), “Ordinance Related to Standards for Conducting Post-Marketing Surveys and Studies on Drugs (MHLW Ministerial Ordinance No.171 dated December 20, 2004)”, and “Guideline on the Implementation Procedures of Post-Marketing Surveillance, etc. for Prescription Drugs (Notification No.1027001 by the Director of the Evaluation and Licensing Division, Pharmaceutical and Food Safety Bureau, MHLW, dated October 27, 2005) in order to confirm the following items for understanding issues and questions and to examine the necessity of a special drug use investigation and a post-marketing clinical study.

(1) Unknown adverse drug reactions (ADRs)

(2) Status of the onset of ADRs under the actual usage conditions of the drug
- Hemorrhagic-related ADRs (especially hemorrhagic-related ADRs in patients weighing ≤50 kg or patients aged ≥75 years)
- Increased liver enzyme(s), including bilirubin
- Efficacy-related events of ischemic stroke, hemorrhagic stroke, non-central nervous system embolism, and myocardial infarction

(3) Factors that may affect the safety and efficacy of the drug

2. PLANNED NUMBER OF PATIENTS

Planned number of patients: 10,000 patients

3. TARGET POPULATION AND DOSAGE REGIMEN

(1) Target population

Patients with non-valvular atrial fibrillation who have received Xarelto for the purpose of prevention of ischemic stroke and systemic embolism

(2) Dosage regimen

To adult patients, usually, 15 mg of rivaroxaban will be administered orally once daily after meal. For patients with renal impairment, the dose will be decreased to 10 mg once daily according to the severity of renal impairment.

4. STUDY METHODS

(1) Target sites

Medical institutions that include Xarelto in their formulary, where the drug is delivered, and where electric data capture (EDC) can be performed will be included in the investigation. Medical representatives shall explain the objectives, target subjects, study methods, etc. of the

investigation to the physicians who will be in charge of the investigation and request the conduct of the investigation to the head of the institution (director, etc.). Then, an agreement in writing will be made with the institution.

(2) Study methods

The "central registration method" using EDC will be introduced in the investigation.

In the EDC system, the physician in charge of the investigation will register the patient by entering the required registration information in the electronic registration form at the timing of initiation of Xarelto within the contracted period of investigation. Patients who have not been treated with Xarelto can be registered in this investigation, and patients who are under treatment with Xarelto initiated at other medical institutions will be excluded. Patients shall be registered by the physician in charge of the investigation within 14 days from the date of initiation defined as Day 0. The registration of patients will be continued until enrollment of the contracted planned number of patients is achieved. The physician in charge of the investigation will enter the required registration information in the EDC system for all the target patients enrolled in the investigation.

The results of this investigation will be entered in the EDC system at 6 months, 1 year, and 2 years and subsequently on an annual basis within the period of investigation.

5. PLANNED STUDY PERIOD

Registration period: release date to March 31, 2015

Investigation period: release date to March 31, 2019

6. STUDY VARIABLES

(1) Observation period

[1] The observation period will normally last 2 years from the initiation of Xarelto.

[2] An outcome investigation for up to 5 years will be conducted after completion of the normal observation period.

[3] If Xarelto is discontinued within 2 years after the initiation of Xarelto, information on medication switching and adverse events (AEs) will be collected as much as possible during the period for up to 30 days after discontinuation of the drug.

(2) Patient information and clinical findings required at registration

1) Patient information

Patient ID, patient initials, date of birth or age, sex, height, body weight, inpatient/outpatient

category, date of initiation of Xarelto, purpose of initiation of Xarelto, presence/absence of previous anticoagulant treatments

2) Clinical findings
Presence/absence of congestive heart failure, presence/absence of hypertension, presence/absence of diabetes mellitus, presence/absence of previous ischemic stroke, presence/absence of previous hemorrhagic stroke, presence/absence of transient ischemic attack, serum creatinine level, PT-INR

(3) Study variables

1) Patient background characteristics
Patient ID, patient initials, date of birth or age, sex, height, body weight, inpatient/outpatient category, presence/absence of renal impairment (disease name), presence/absence of hepatic function disorder (Child-Pugh classification, disease name), presence/absence of other underlying conditions, predispositions of hypersensitivity, history of smoking

2) History of the primary disease
Purpose of initiation of Xarelto, date of the initial onset of atrial fibrillation, disease type of non-valvular atrial fibrillation

3) Previous medical treatments
History of anti-coagulation therapy and anti-platelet therapy within 30 days, names of drugs, route of administration, daily dose (unit), treatment period, reason(s) for switching to (adding of) Xarelto

4) Status of the treatment with Xarelto
Start date (start date after switching medication or start date of interruption), daily dose, status of interruption (reason[s] for interruption, other medical treatments during interruption, onset of new AEs during interruption), reason[s] for the change in the dose, drug compliance, status of administration at completion of the observation period, end date of administration of Xarelto, presence/absence of switching to anticoagulants (name[s] of drug[s], route of administration, (initial) daily dose, dosing method, start date), onset of AEs within 30 days after withdrawal from Xarelto

5) Patient outcome
The patient outcome shall be entered. In the cases of death, the cause of death shall be entered

in detail in the column of AEs.

6) Laboratory tests

If the following test items have been examined, the values shall be entered. If a laboratory value after initiation of the treatment with Xarelto is considered to correspond to an AE, its details shall be entered in the column of AEs.

[1] Blood pressure (systolic/diastolic), body weight

[2] Hematology: PT-INR, prothrombin time, prothrombin concentration (activity), activated partial thromboplastin time (APTT), fibrinogen, FDP, D-dimer, hemoglobin, platelets

[3] Blood biochemistry: creatinine, creatinine clearance (by automatic calculation), BUN, potassium, AST, ALT, gamma-GTP, ALP, total bilirubin, total cholesterol, CRP

7) Efficacy-related events

Efficacy-related events: ischemic stroke, hemorrhagic stroke, non-central nervous system embolism, and myocardial infarction

Non-central nervous system embolism: acute circulatory failure in the non-cerebral blood vessels associated with clinical findings or diagnostic images of arterial embolism with no other possible cause (e.g., trauma, atherosclerosis, use of devices, excluding pulmonary embolism and myocardial infarction)

The seriousness of the events shall be assessed according to the descriptions in "8) Adverse events."

The following information shall be entered regarding the efficacy-related events after initiation of Xarelto.

- Presence/absence of efficacy-related events, name(s) of the efficacy-related event(s), site of stroke, site of non-central nervous system embolism, date of onset, outcome, date of outcome, seriousness, rationale for the seriousness, treatment provided, other medication provided, causality with Xarelto, factors other than Xarelto

8) Adverse events (AEs)

Adverse event: any untoward medical event in patients receiving a drug regardless of the causality with the drug. An AE is therefore any unfavorable and unintended sign (e.g., abnormal laboratory test value), symptom, or disease with a temporal correlation with the use of the drug, regardless of presence/absence of causality with the drug.

Hemorrhagic-related event: hemorrhage with a clinically (macroscopically, diagnostic image-based, or laboratory test-based) obvious source of hemorrhage

Significant hemorrhagic-related event: clinically obvious hemorrhage associated with the following:

- A decrease in hemoglobin by 2 g/dL or greater
- A need for blood transfusion (packed red blood cells or whole blood) of 2 units or greater
- Hemorrhage in important organs (e.g., intracranial hemorrhage, intraspinal hemorrhage, intraocular hemorrhage, pericardial hemorrhage, intra-articular hemorrhage, intramuscular hemorrhage associated with compartment syndrome, peritoneal hemorrhage)
- Fatal hemorrhage

Seriousness: The following shall be assessed as "serious."

- Death due to the AE
- Life-threatening AEs
- Hospitalization or prolonged hospitalization because of treatment of the AE
- Permanent or significant disability/incapacity
- AEs that may cause congenital anomaly
- Medically significant conditions

The following information shall be entered regarding the AEs (excluding efficacy-related events) after initiation of Xarelto.

- Presence/absence of AEs, name(s) of the AE(s), correspondence with a significant hemorrhagic-related event, date of onset, outcome, date of outcome, seriousness, rationale for the seriousness, treatment provided, other medication provided, causality with Xarelto, factors other than Xarelto

9) Clinical laboratory parameters related to AEs

Name(s) of the parameter(s), name(s) of the relevant efficacy-related events/AEs, test values, and other information shall be entered if there are any test values related to the AE(s). However, the parameters specified in "6) Laboratory test" shall be excluded.

10) Concomitant drugs

Presence/absence of concomitant drugs, name(s) of the concomitant drug(s), route of administration, daily dose (unit), duration of the treatment, purpose(s) of use

11) Non-drug therapy (therapies) for the treatment of atrial fibrillation

Presence/absence of non-drug therapy (therapies) for the treatment of atrial fibrillation,

name(s) of the non-drug therapy (therapies) for the treatment of atrial fibrillation, date(s) of the therapy (therapies)

12) Date of the last observation

The most recent date of contact with the patient (treatment/physical examination/communication) or date of the patient's death

13) Outcome investigation

The status of treatment with Xarelto (presence/absence of ongoing treatment and reason[s] for discontinuation/withdrawal), patient outcome, efficacy-related event(s), serious AE(s), and date of the last observation shall be entered.

(4) Focused investigation items

"Hemorrhage," which has been defined as an important identified risk in the risk management plan in Japan, and "increased liver enzyme(s)," which has (have) been defined as an important potential risk in the risk management plan in Japan, will be the investigation items of focus.

7. ESTABLISHMENT OF THE POST-MARKETING SURVEILLANCE COMMITTEE

The information to be collected in this investigation will be used not only for internal review of safety and efficacy but also for review of the results by the committee members listed below. The results of the review shall be published by appropriate methods such as publication as a research paper in order to ensure proper use of the drug.

Chairperson:	Satoshi Ogawa, IUHW Mita Hospital
Committee members:	Takanori Ikeda, Toho University Omori Medical Center
	Takanari Kitazono, Faculty of Medical Sciences, Kyushu University
	Jyoji Nakagawara, Nakamura Memorial Hospital
	Kazuo Minematsu, National Cerebral and Cardiovascular Center
	Susumu Miyamoto, Department of Neurosurgery, Kyoto University Hospital
	Yuji Murakawa, Teikyo University Hospital Mizonokuchi

8. CONTACT FOR INQUIRIES

(1) Contact for inquiries about the details of the investigation

PMS, Pharmacovigilance Division, Medical Affairs Department, Bayer Yakuhin Ltd.

Phone: 06-6133-6300 Fax: 06-6344-2264

(2) Contact for inquiries about the EDC system

PostMaNet, Fujitsu FIP Corporation

Phone: 0120-002-593 Inquiry code: L0292

Business hours: 9:00 to 21:00 (except Saturdays/Sundays/national holidays)
