# Supplementary material for: Real-world safety and effectiveness of rivaroxaban using Japan-specific dosage during long-term follow-up in patients with atrial fibrillation: XAPASS
Source: PLoS One. 2021 Jun 11;16(6):e0251325. doi: 10.1371/journal.pone.0251325 (PMC8195353; doi:10.1371/journal.pone.0251325)
Supplement: S2 File — (PDF) [file pone.0251325.s012.pdf]

イグザレルト錠
特定使用成績調査（SPAF）
実施要綱

－非弁膜症性心房細動患者における虚血性脳卒中及び全身性塞栓症の発症抑制－

バイエル薬品株式会社

作成年月日：平成 24 年 1 月 20 日

目次

1. 調査の目的 1

2. 調査予定症例数 1

3. 調査対象患者及び用法・用量 1

4. 調査方法 1

5. 調査の実施予定期間 2

6. 調査を行う事項等 2

7. PMS 委員会の設置 4

8. 問い合わせ先 5

1. 調査の目的

薬事法第 14 条の 4（再審査）の規定、「医薬品の製造販売後の調査及び試験の実施の基準に関する省令（平成 16 年 12 月 20 日付厚生労働省告示第 171 号)」及び「医療用医薬品の製造販売後調査等の実施方法に関するガイドラインについて（平成 17 年 10 月 27 日付薬食審査発第 1027001 号)」等に基づいて以下の事項を確認し、問題点、疑問点等を把握するとともに、特定使用成績調査及び製造販売後臨床試験の必要性の有無を検討する。

（１）未知の副作用

（２）医薬品の使用実態下における副作用の発現状況の把握

・出血性の副作用（特に、体重 50kg 以下の症例及び 75 歳以上の症例での出血性の副作用）

・肝酵素（ビリルビンを含む）上昇の副作用

・虚血性脳卒中、出血性脳卒中、非中枢神経系塞栓症、心筋梗塞の有効性イベント

（３）安全性又は有効性等に影響を与えると考えられる要因

2. 調査予定症例数

調査予定症例数：10,000 症例

3. 調査対象患者及び用法･用量

(1)調査対象患者

非弁膜症性心房細動患者における虚血性脳卒中及び全身性塞栓症の発症抑制を目的として本剤を投与された患者

(2)用法･用量

通常、成人にはリバーロキサバンとして、15 ㎎を 1 日 1 回食後に経口投与する。なお、腎障害のある患者に対しては、腎機能の程度に応じて 10 ㎎ 1 日 1 回に減量する。

4. 調査方法

(1)調査対象医療機関

本剤が採用・納入された、かつ EDC（Electronic Data Capture）での調査が可能な医療機関を対象とし、医薬情報担当者が対象施設の調査担当医師に対して、本調査の目的、調査対象、調査方法等を説明し、施設長（院長等）に対して依頼を行い、文書で契約を交わす。

(2)調査方法

調査方法は、EDC を用いた「中央登録方式」とする。

調査担当医師は、契約書の調査期間内で対象患者に本剤を投与した時点で、EDC の登録画面に必要事項を入力し、登録を行う。登録可能な患者は、本剤未治療例とし、他院で本剤が投与開始され、継続投与されている患者は除外する。登録は、原則として投与日を 0 日として 14 日以内に調査担当医師が行う。対象患者の登録は、登録期間内で契約書の予定症例数に至るまで行う。

調査担当医師は、登録した全ての対象患者について必要事項を EDC に入力する。

本調査の調査結果は、6 ヵ月、1 年、2 年経過時点で EDC に入力する。それ以降は、調査期間内で 1 年経過時点毎に EDC に入力する。

5. 調査の実施予定期間

登録期間：販売開始日～平成 27 年 3 月 31 日

調査期間：販売開始日～平成 31 年 3 月 31 日

6. 調査を行う事項等

（1）観察期間

①標準観察期間は、本剤投与開始後 2 年間とする。

②標準観察期間終了後は、最長 5 年間の予後調査を行う。

③本剤投与開始後 2 年以内に本剤の投与終了の場合、本剤の投与終了後 30 日間を最長として、可能な限り他剤への切替え状況及び有害事象の確認を行う。

（2）登録時における患者情報及び臨床所見

1）患者情報

患者識別番号、患者イニシャル、生年月日（年齢）、性別、身長、体重、受診区分、イグザレルト錠投与開始日、イグザレルト錠投与目的、他の抗凝固薬からの切替え

2）臨床所見

うっ血性心不全の有無、高血圧症の有無、糖尿病の有無、虚血性脳卒中の既往、出血性脳卒中の既往、一過性脳虚血発作の既往、血清クレアチニン値、PT-INR

（3）調査を行う事項

1）患者背景

患者識別番号、患者イニシャル、生年月日（年齢）、性別、身長、体重、受診区分、腎機能障害（疾患名）、肝機能障害（Child-Pugh 分類、疾患名）、その他の基礎疾患、過敏性素因、喫煙歴

2）原疾患病歴

イグザレルト錠投与目的、心房細動初発年月、非弁膜症性心房細動の病型

3）前治療歴

本剤投与前 30 日間の抗凝固療法・抗血小板療法歴、薬剤名、投与経路、1 日投与量（単位）、治療期間、本剤への切替え（追加投与）の理由

4）本剤の投与状況

開始日（変更後の投与開始日又は休薬開始日）、1 日投与量、休薬期間の状況（休薬の理由、休薬期間中の他薬剤処置、休薬期間中の新たな有害事象の発現）、投与量変更の理由、服薬コンプライアンス、観察期間終了時点の投与状況、投与終了日、抗凝固薬への切替え（薬剤名、投与経路、1 日投与量（初回）、投与方法、投与開始日）、本剤投与終了後 30 日以内の有害事象の発現

5）患者転帰

患者転帰を入力する。なお、死亡の場合は、有害事象欄に死因の詳細を入力する。

6）臨床検査

以下の検査項目が測定されていた場合、検査値を入力する。有害事象に該当すると判断された項目は有害事象欄に詳細を入力する。

① 血圧（収縮期血圧／拡張期血圧）、体重

② 血液学検査：PT-INR、プロトロンビン時間、プロトロンビン濃度（活性）、活性化部分トロンボプラスチン時間（APTT）、フィブリノゲン、FDP、D ダイマー、ヘモグロビン、血小板

③ 血液生化学検査：クレアチニン、クレアチニンクリアランス（自動計算値）、BUN、カリウム、AST、ALT、γ-GTP、ALP、総ビリルビン、総コレステロール、CRP

7）有効性イベント

有効性イベント：虚血性脳卒中、出血性脳卒中、非中枢神経系塞栓症及び心筋梗塞とする。

非中枢神経系塞栓症：脳血管以外の血管に生じた、他に考えられる原因（例、外傷、アテローム性動脈硬化、器具使用）のない、動脈閉塞の臨床的所見若しくは画像所見を伴う急性血行不全とする（肺塞栓症、心筋梗塞は含まない）。

なお、重篤度の判定は、８）有害事象の記載に従う。

本剤投与開始以降に出現した有効性イベントについて、以下の内容を入力する。

有効性イベントの発現有無、有効性イベント名、脳卒中部位、非中枢神経系塞栓部位、発現日、転帰、転帰日、重篤度、重篤理由、処置、他薬剤処置、本剤との因果関係、本剤以外の要因

8）有害事象

有害事象：医薬品が投与された患者に生じたあらゆる好ましくない医療上の出来事であり、必ずしも当該医薬品の投与と因果関係があるもののみを示すわけではない。すなわち、有害事象とは、医薬品の使用と時間的に関連のある、あらゆる好ましくない、意図しない徴候（例えば、臨床検査値の異常）、症状または疾患のことであり当該医薬品との因果関係の有無は問わない。

出血事象：臨床的（肉眼的、画像所見的、臨床検査的）に出血源が明らかな出血とする。

重大な出血事象：下記を伴う臨床的に明らかな出血とする。

- 2g/dL 以上のヘモグロビン量の低下を伴う出血
- 2 単位以上の輸血（濃厚赤血球又は全血）が必要な出血
- 重要な臓器における出血；頭蓋内出血、髄腔内出血、眼内出血、心嚢内出血、関節内出血、コンパートメント症候群を伴う筋肉内出血、後腹膜出血等
- 死因となった出血

重篤度：重篤とは、以下に該当するものとする。

- 本有害事象による死亡
- 生命を脅かすもの
- 本有害事象の治療のための入院または入院延長
- 永続的または顕著な障害・機能不全
- 先天異常を来たすもの
- 医学的に重大な状態

本剤投与開始以降に出現した有害事象（有効性イベントを除く）について、以下の内容を入力する。

有害事象の発現有無、有害事象名、出血事象の該当有無、重大な出血事象の該当有無、発現日、転帰、転帰日、重篤度、重篤理由、処置、他薬剤処置、本剤との因果関係、本剤以外の要因

9）有害事象に関する臨床検査項目

有害事象に関連する検査値がある場合、項目名、関連する有効性イベント名／有害事象名、検査値等を入力する。ただし、６）臨床検査に規定されている項目を除く。

10）併用薬剤

併用薬剤の有無、併用薬剤名、投与経路、１日投与量（単位）、投与期間、使用目的

11）心房細動に対する非薬物療法

心房細動に対する非薬物療法の有無、心房細動に対する非薬物療法名、施行日

12）最終観察日

患者とコンタクト（診療・診察・連絡受信）した直近の日又は患者死亡日

13）予後調査

本剤の投与状況（継続の有無、中止・脱落理由）、患者転帰、有効性イベント、重篤な有害事象及び最終観察日を入力する。

（4）重点調査事項

本邦のリスクマネジメントプランにおいて重要な特定されたリスクとされた“出血”並びに本邦のリスクマネジメントプランにおいて重要な潜在的なリスクとされた“肝酵素（ビリルビンを含む）の上昇”を重点調査項目とする。

7. PMS 委員会の設置

本調査により収集された情報は、社内にて安全性・有効性を検討するのみならず、以下の委員により調査結果を検討する機会を設ける。検討結果は、適正使用確保のため、論文化等の適切な方法により公表する。

委員長：小川　　聡　先生　国際医療福祉大学三田病院
委員：池田　隆徳　先生　東邦大学医療センター大森病院
北園　孝成　先生　九州大学大学院医学研究院
中川原譲二　先生　中村記念病院
峰松　一夫　先生　国立循環器病研究センター
宮本　　享　先生　京都大学医学部附属病院
村川　裕二　先生　帝京大学医学部附属溝口病院　（五十音順）

8.　問い合わせ先

（1）調査内容に関する問い合わせ先

バイエル薬品株式会社

メディカルアフェアーズ本部 ファーマコビジランス PMS

電話：06-6133-6300　FAX：06-6344-2264

（2）EDC システムに関する問い合わせ先

富士通エフ・アイ・ピー株式会社　PostMaNet CSD

電話：0120-002593　お問合せコード：L0292

受付時間：9:00～21:00（土日祝祭日は除く）
