# Supplementary material for: Real-world safety and effectiveness of rivaroxaban using Japan-specific dosage during long-term follow-up in patients with atrial fibrillation: XAPASS
Source: PLoS One. 2021 Jun 11;16(6):e0251325. doi: 10.1371/journal.pone.0251325 (PMC8195353; doi:10.1371/journal.pone.0251325)
Supplement: Attachment — Submitted filename: Japenese study.docx [file pone.0251325.s013.docx]

**Reviewer comments:**

**#1: Methods:**

Please mention inclusion and exclusion criteria

**#2** : **Study limitations:**

Please mention about lack of generalized results as limited to only Japanese and also limited to low to intermediate risk CHADVASc score.

**#3:** Elaborate the reasons why do you have similar efficacy and safety issues with usage of low dose comparted to recommended dose? Also, it seems like most of the patients have low to intermediate CHADVASs score which could be the limitation for real world practice.

**#4:** Overall, it is a good prospective study with a long term results and authors have done a great job!
